# HectoSTAR μLED Optoelectrodes for Large‐Scale, High‐Precision In Vivo Opto‐Electrophysiology

**DOI:** 10.1002/advs.202105414

**Published:** 2022-04-22

**Authors:** Mihály Vöröslakos, Kanghwan Kim, Nathan Slager, Eunah Ko, Sungjin Oh, Saman S. Parizi, Blake Hendrix, John P. Seymour, Kensall D. Wise, György Buzsáki, Antonio Fernández‐Ruiz, Euisik Yoon

**Affiliations:** ^1^ Department of Electrical Engineering and Computer Science University of Michigan Ann Arbor MI 48109 USA; ^2^ Neuroscience Institute Langone Medical Center New York University New York NY 10016 USA; ^3^ Center for BioMicrosystems Brain Science Institute Korea Institute of Science and Technology Seoul 02792 South Korea; ^4^ Department of Neurosurgery University of Texas Health Science Center Houston TX 77030 USA; ^5^ Department of Neurobiology and Behavior Cornell University Ithaca NY 14853 USA; ^6^ Department of Biomedical Engineering University of Michigan Ann Arbor MI 48109 USA; ^7^ Center for Nanomedicine Institute for Basic Science (IBS) and Graduate Program of Nano Biomedical Engineering (Nano BME) Advanced Science Institute Yonsei University Seoul 03722 South Korea

**Keywords:** μLED, large‐scale optoelectrophysiology, neural probe, neuronal ensembles, optogenetics

## Abstract

Dynamic interactions within and across brain areas underlie behavioral and cognitive functions. To understand the basis of these processes, the activities of distributed local circuits inside the brain of a behaving animal must be synchronously recorded while the inputs to these circuits are precisely manipulated. Even though recent technological advances have enabled such large‐scale recording capabilities, the development of the high‐spatiotemporal‐resolution and large‐scale modulation techniques to accompany those recordings has lagged. A novel neural probe is presented in this work that enables simultaneous electrical monitoring and optogenetic manipulation of deep neuronal circuits at large scales with a high spatiotemporal resolution. The “hectoSTAR” micro‐light‐emitting‐diode (μLED) optoelectrode features 256 recording electrodes and 128 stimulation μLEDs monolithically integrated on the surface of its four 30‐µm thick silicon micro‐needle shanks, covering a large volume with 1.3‐mm × 0.9‐mm cross‐sectional area located as deep as 6 mm inside the brain. The use of this device in behaving mice for dissecting long‐distance network interactions across cortical layers and hippocampal regions is demonstrated. The recording‐and‐stimulation capabilities hectoSTAR μLED optoelectrodes enables will open up new possibilities for the cellular and circuit‐based investigation of brain functions in behaving animals.

## Graphical abstract

A novel neural probe that enables simultaneous electrophysiological recording and optogenetic manipulation of deep neuronal circuits at large scales with a high spatiotemporal resolution is presented. The hectoSTAR micro‐light‐emitting‐diode (μLED) optoelectrode, featuring 256 recording electrodes and 128 stimulation μLEDs densely integrated on the tip of four micro‐needles, allows for cellular and circuit‐based brain mapping in behaving animals.

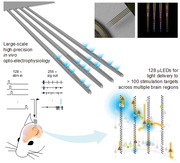

## 1 Introduction

The understanding of the neural basis of behavioral and cognitive functions begins from the observation of how the communication among neuronal ensembles across different brain areas occur. Important advances have been achieved by recording and manipulating neural activity in in vitro preparations, in particular regarding the detailed synaptic organization of neuronal microcircuits via observation of sub‐threshold intracellular activities such as post‐synaptic membrane potentials.^[^ ^1^, ^2^ ^]^ However, in order to understand how neuronal activities give rise to complex brain functions, it is necessary to monitor and control the activity of neurons in behaving animals at high spatiotemporal resolutions. Recent technical developments have provided new methods for such large‐scale, in vivo recordings across brain areas with single‐cell resolution either using electrophysiological^[^ ^3^, ^4^, ^5^, ^6^, ^7^ ^]^ or imaging^[^ ^8^, ^9^, ^10^ ^]^ approaches. These developments have enabled important advances in our understanding of the neural mechanisms of behavior, moving from a single brain area‐centric perspective to a dynamically interacting distributed circuits view.^[^ ^11^, ^12^ ^]^ A deeper understanding of how specific neural patterns, or the activity of individual neurons give rise to behavioral and cognitive functions, however, requires an additional capability to precisely perturb the activity of the specific neuronal subset. A powerful perturbation method is optogenetics,^[^ ^13^, ^14^, ^15^, ^16^, ^17^, ^18^, ^19^ ^]^ and the development of optoelectrodes^[^ ^20^, ^21^, ^22^, ^23^, ^24^, ^25^, ^26^, ^27^ ^]^ paved the way forward to a more precise interrogation of neural circuit function.

A common challenge for combining electrophysiological recordings with optogenetic manipulations in behaving animals is the delivery of light to deep brain structures with high spatial resolution, ideally to individual cells. Two well‐known approaches for light delivery are integrating light‐guide structures on an electrode array^[^ ^22^, ^23^, ^24^, ^25^, ^26^ ^]^ and optics‐assisted multi‐photon stimulation.^[^ ^28^, ^29^, ^30^, ^31^, ^32^ ^]^ While each of these approaches provides unique advantages, neither of them is suitable for applications that require large‐scale, high‐resolution probing of deep neural structures. These approaches can neither provide stimulation at sufficiently high resolution (light‐guide approach) nor deliver light into the deep brain (multi‐photon approach). One promising, yet challenging, method for light delivery is the use of miniature light sources, such as micro‐light‐emitting‐diodes (μLEDs), directly located at the target region, so that multiple light sources can selectively modulate neurons whose activities are actively monitored by the electrodes.^[^ ^27^ ^]^ However, due to the existence of large stimulation artifacts^[^ ^27^, ^33^ ^]^ and the limited surface area on the device, the μLED optoelectrodes could not be scaled up to enable sampling from a large volume of the brain.

In this work, we report a novel optoelectrode that provides the capability of large‐scale neuronal activity recordings, combined with the ability to precisely stimulate neurons located at more than a hundred (hecto‐) Stimulation Targets Across Regions (STAR) using monolithically integrated μLEDs. The hectoSTAR μLED optoelectrode features several engineering innovations in the nanofabrication and assembly of integrated components to provide an order of magnitude increase compared to previous devices^[^ ^27^, ^33^ ^]^ in the number of recording and stimulation sites with a three‐fold higher density without any compensation of the recording and the stimulation performances. The engineering innovations include the multi‐metal‐layer architecture for the mitigation of stimulation artifacts, the high‐resolution metal patterning and higher‐density integration of optoelectronic components, and the use of a microfabricated interposer for the area‐efficient packaging of a high‐channel‐count product. Thanks to these innovations, 256 recording sites and 128 μLEDs on the hectoSTAR μLED optoelectrode span a 900 × 1300 µm brain area which allows the investigation of interactions across brain areas. In addition to the optoelectrode, we introduce a custom‐developed Field‐Programmable‐Gate‐Array (FPGA)‐based controller for the independent manipulation of each μLED with arbitrarily defined pulse shapes and dynamics. With the hectoSTAR μLED optoelectrode and the controller, we conducted the first‐of‐a‐kind experiment in behaving mice in order to address questions that were not tractable before with existing technology. As a demonstration, we present the utility of the hectoSTAR optoelectrode for dissecting network interactions across cortical layers and hippocampal regions.

## 2 Results

### 2.1 HectoSTAR μLED Optoelectrode Allows for Large‐Scale In Vivo Opto‐Electrophysiology

**Figure 1:**
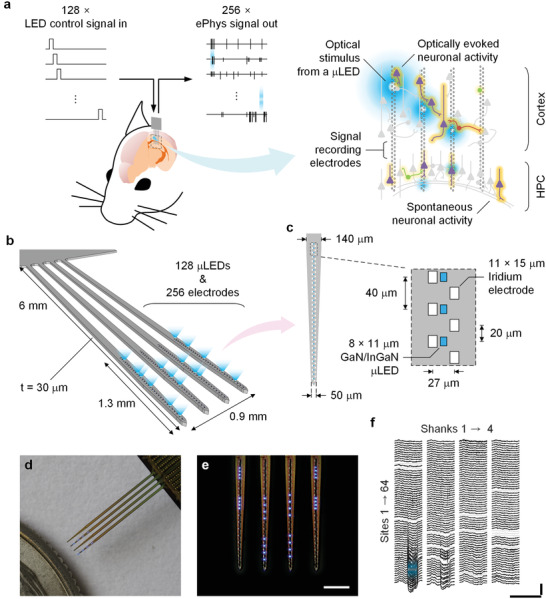
HectoSTAR μLED optoelectrode enables high‐precision and large scale deep‐brain opto‐electrophysiology. a) A conceptual drawing of a large‐scale in vivo opto‐electrophysiology experiment conducted using a hectoSTAR μLED optoelectrode. HectoSTAR μLED optoelectrode can deliver arbitrary optical stimulation patterns to multiple deep‐brain locations within a large area, spanning from the whole cortical layers to CA1 region of dorsal hippocampus, while simultaneously recording single units and local‐field potentials from the region. Brain schematic (left) is in scale with the length of hectoSTAR optoelectrode. Grey objects indicate non‐active neurons and colored objects active neurons. White rectangles show the locations of the recording sites, and the blue glowing spots represent an example stimulation pattern generated from multiple μLEDs. b) A 3D model of a hectoSTAR μLED optoelectrode generating a complex optical stimulation pattern. The hectoSTAR μLED optoelectrode has four, 6‐mm long and 30‐µm thick shanks, and the pitch between two neighboring shanks is 300 µm. Each shank can record and stimulate across 1.3 mm along the dorsoventral axis. c) Detailed schematic diagram of a tip of a shank. The inset shows the dimensions of and the distances between iridium electrodes (recording sites, 64 per shank) and blue‐light‐emitting GaN/InGaN μLEDs (stimulation sites, 32 per shank). Recording sites are arranged in a “staggered” configuration with less than 40‐µm center‐to‐center pitch. μLEDs are located along the center of the optoelectrode shank with 40 µm center‐to‐center pitch. d,e) Microphotographs of a fabricated hectoSTAR μLED optoelectrode. A packaged hectoSTAR optoelectrode is photographed next to a U. S. quarter in (d). Scale bar is 300 µm long in (e). Note blue light being generated from active μLEDs. f) Example of local field potential recordings from a hectoSTAR optoelectrode, in which induced response resulting from an optical stimulus provided from a single μLED (blue) is shown. Grey traces are non‐functional channels.

The hectoSTAR μLED optoelectrode was designed to enable high‐resolution, large‐scale opto‐electrophysiology. More specifically, the optoelectrode was designed to record extracellular spikes and local‐field potentials (LFPs) from a large brain area and deliver optical stimuli to selected neurons within the regions it can record, while recording each neuron's spikes with multiple electrodes. The probe geometry was designed to optimally record from 2D laminar structures such as several cortical columns or hippocampal subregions in rodents (**Figure** **1**a). A hectoSTAR μLED optoelectrode contains 256 electrodes and 128 μLEDs monolithically integrated on its four shanks, each of which is 6‐mm long. The recording and stimulation sites span a brain area as large as 1.17 mm^2^ (900 µm × 1300 µm; Figure 1b). At the same time, the cross‐sectional area of each shank of the optoelectrode was minimized to reduce the acute damage induced in the tissue during insertion (Supporting Information).

As shown in Figure 1c, each shank of an optoelectrode contains 64 electrodes and 32 LEDs on its tip. Two rows of iridium electrodes, each of which contains thirty‐two small (11 µm × 15 µm) electrodes, are located along the center of the shank. The vertical distance between two adjacent electrodes on each column and the horizontal distance between the columns were chosen to be 40 and 27 µm, respectively, so that the distance between any two adjacent electrodes is no >40 µm. As an electrode located <60 µm away from the soma (or the axon) of a neuron can reliably record action potentials generated from the neuron,^[^ ^34^, ^35^, ^36^ ^]^ the dense electrode configuration allows multiple electrodes to simultaneously record the action potentials (spikes) of individual neurons facilitating spike sorting (Figure S1, Supporting Information).^[^ ^37^ ^]^ μLEDs with dimensions comparable to the size of a neuronal soma (8 µm × 15 µm) are located at the center of the shank, allowing a precise, co‐localized optical stimulation of the neurons recorded by nearby electrodes. The vertical distance between two adjacent LEDs was set as 40 µm, identical to the vertical pitch of the electrodes on each column, so that a neuron whose activity is being recorded can be illuminated with at least one μLED. As shown in Figure 1d and e, blue light is emitted from each μLED, and its spectrum (*λ*_peak_ ≈ 470 nm) is ideal for the activation of channelrhodopsin‐2 opsins. The on‐and‐off timing and the intensity of the optical stimulation each LED generates can be independently controlled so that any intricate stimulation patterns, an example of which is shown in Figure 1d, can be generated at any moment, either pre‐defined or on‐the‐fly through an open‐loop setup, during an experiment (Movie S1, Supporting Information).

Example neural signals recorded from a hectoSTAR μLED optoelectrode, shown in Figure 1f, clearly demonstrate the optoelectrode's recording and stimulation capability. As shown in Figure 1f, neuronal activities occurring at different locations on several deep‐brain regions—here cortical layers and hippocampal CA1—can be easily captured with a single insertion of the optoelectrode. In the traces of large‐channel‐count recording are examples of population activities (high‐frequency fluctuation recorded from the sites in CA1 and intermittent “dips” in recorded from sites in the cortex), all resulting from a precise optical stimulation of a small brain region (CA1 pyramidal layer, indicated with a blue highlight).

### 2.2 High‐Density, Large‐Scale Integration of μLEDs and Electrodes on a Minimal‐Form‐Factor Platform

**Figure 2:**
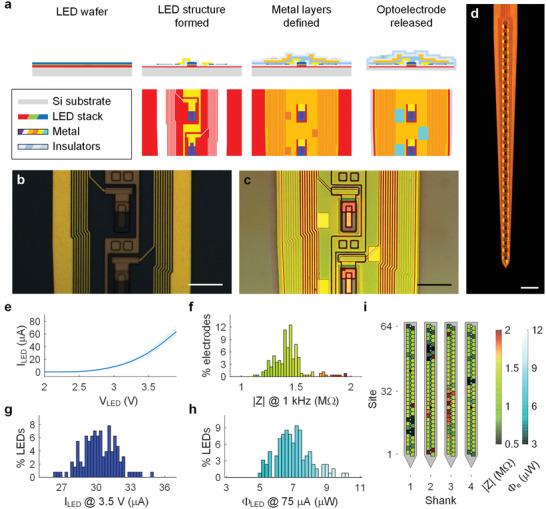
Monolithically integrated components of hectoSTAR μLED optoelectrodes are ideal for high‐precision in vivo opto‐electrophysiology. a) Snapshots of an imaginary cross‐section view (top row) and a top view (bottom row) of a part of the hectoSTAR μLED optoelectrode shank after each of three key fabrication steps. In the cross‐sectional views, the scales along *x* (width) and *y* (thickness) axes are not identical with each other for illustration purpose. Microphotographs of the surface of a shank b) after formation of the μLED structures and c) after the formation of the interconnects for the signal recording electrodes. Both scale bars are 20 µm long. d) Microphotograph of a shank of a released hectoSTAR μLED optoelectrode. Scale bar is 100 µm long. e) Voltage–current relationship of the μLEDs on a typical hectoSTAR μLED optoelectrode. The solid line indicates the median, and the shadowed area the separation between the minimum and the maximum currents at each forward bias voltage. f–h) Histograms showing distributions of electrical and optical characteristics of the electrodes and the LEDs on a typical hectoSTAR μLED optoelectrode. The impedance magnitude of the electrodes measured at 1 kHz, the current of the LEDs with 3.5 V forward biased voltage and the radiant flux from the LEDs with 75‐µA current are shown in (f), (g), and (h), respectively. i) Heatmaps showing the spatial distribution of the electrode impedance and the LED radiant flux on a typical optoelectrode.

The hectoSTAR optoelectrodes incorporates heterogeneous components on its surface at both high density and large scale, and the integration is realized by advanced fine‐pitch metal patterning together with multi‐layer metal stacking. In the μLED optoelectrodes that had been developed to date,^[^ ^27^, ^33^ ^]^ the number of integrated μLEDs and electrodes per each shank has been limited to a small number (≈10 total) due to the large space that the metal traces occupy on the surface (≈4 µm per each trace). Significant amount of engineering development and optimization was undertaken to break through limits in the existing μLED optoelectrode fabrication process so that the optoelectrode can accommodate a number of μLEDs and electrodes at an approximately three‐fold higher density within a given constraint of shank dimensions to mitigate tissue damage. **Figure** **2**a shows schematic diagrams of the cross‐section of a hectoSTAR μLED optoelectrode after a few key steps of the fabrication process. The interconnects for both LED drive signals and recorded neural signals are formed at 0.7‐µm half‐pitch, which can provide ≈700 metal‐trace lines per millimeter. With these extremely fine‐pitched metal traces, the narrow shank profile (tapering to 50 µm at the bottommost μLED, 140 µm above the top μLED) could be achieved.

An innovative patterning technique was introduced to enable the formation of high‐density metal traces without the use of expensive e‐beam or EUV lithography. Both the μLED and recording traces were built of 100‐nm thick gold layers, and patterns with sub‐micron features were formed on each layer using lift‐off process following an i‐line photolithography step using a step‐and‐repeat wafer exposure tool. A bi‐layer resist stack with a contrast‐enhancing top layer^[^ ^38^, ^39^, ^40^ ^]^ was used for the reliable formation of the ideal retrograde sidewall profile (Figure S2, Supporting Information) uniformly across a whole 4‐inch wafer. One hundred‐nanometer thick gold (90 nm Au on 10 nm Ti) layer was deposited over the resist sacrificial layer using electron‐beam evaporation, and then lift‐off patterned in a warm bath of N‐Methyl‐2‐pyrrolidone(NMP)‐based solvent. Figure 2b,c show the microphotographs of a hectoSTAR μLED optoelectrode shank after LED formation and electrode definition steps, respectively, illustrating the reliable formation of the submicron interconnects on both metal layers.

Despite the high‐density integration of heterogeneous components, the hectoSTAR μLED optoelectrodes exhibit excellent performance comparable to those of previously reported low‐LED‐density optoelectrodes, supporting its reliable in vivo operation. We characterized the electrical and optical properties of the fabricated hectoSTAR μLED optoelectrodes and confirmed their suitability for in vivo opto‐electrophysiology experiments (Figure 2e–i). A typical μLED allows 1 µA of current when biased at 2.56 ± 0.04 V, and 30.3 ± 1.52 µA at 3.5 V (both mean ± SD, *n* = 121), respectively. The maximum current was set at 75 µA, the recommended maximum current for the safe continuous operation of the LEDs given the dimensions of the interconnects.^[^ ^41^ ^]^ At 75 µA, the μLED generates 7.03 ± 1.05 µW of radiant flux (mean ± SD, *n* = 121) which is equivalent to ≈60 mW cm^−2^ at the surface of the μLED. Most electrodes had impedances between 500 kΩ and 2 MΩ at 1 kHz (Figure 2f), and the median impedance of these electrodes was 1.40 MΩ. The performance of LEDs and the electrode impedance showed narrow distributions (Figure 2e–h), and neither had any correlation with the location of the μLEDs and/or the electrodes on the optoelectrode shank (Figure 2i). The electrode impedances are sufficiently low for the required multiplexed extracellular electrophysiology^[^ ^42^ ^]^ and the LEDs can efficiently generate more than sufficient light.^[^ ^26^, ^27^ ^]^

Results from finite‐element‐method (FEM) based simulations further validated the reliable operation of the hectoSTAR μLED optoelectrodes suitable for in vivo opto‐electrophysiology. Some important device characteristics are difficult to directly measure but can be accurately estimated from simulations, such as the crosstalk between the recorded electrical signals, the illumination profile of a μLED, and tissue heating due to μLED operation. First, the combined electrostatic and circuit simulation of a hectoSTAR μLED optoelectrode (Figure S3a–c, Supporting Information) showed that the voltage signal reaching the electrode is reliably recorded with a minimal loss (<1 dB) and the crosstalk from neighboring channels (< –88 dB) is negligible over the frequency band of physiologically relevant signals (0.1 Hz ≤ f ≤ 10 kHz, Figure S2d, Supporting Information). The simulated irradiance profile resulting from illumination of a μLED showed that the illumination volume (where *Φ*_e_ < 0.1 mW mm^−2^) is confined to a close vicinity of the LED and is within a nearby electrode (Figure S4a, Supporting Information). Finally, simulation of tissue heating confirmed that the temperature increase of the tissue is not >0.6 °C when a μLED is driven at the maximum safe power (*W*_elec_ = 4 V × 75 µA = 300 µW, Figure S4b,c, Supporting Information). All these results indicated that the hectoSTAR optoelectrode can safely operate at high‐spatiotemporal‐resolution and high‐precision in vivo opto‐electrophysiology.

### 2.3 Independent Control of μLEDs for Arbitrarily Patterned Optical Micro‐Stimulation

**Figure 3:**
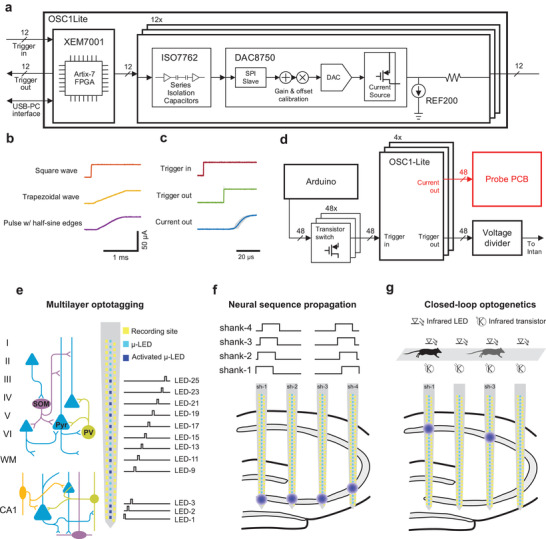
Independent, highly‐multiplexed control of μLEDs using OSC1Lite. a) Schematic diagram of an OSC1Lite. All the commercial off‐the‐shelf integrated circuit (IC) components for each current output channel are shown. b) Snippets of example current waveforms generated from an OSC1Lite channel. Thanks to the fast sampling rate (≈60 kS s^−1^) and the arbitrary waveform generation feature of the controller software, the shape of the current pulse's rising edge can be easily modified into different shapes. Note negligible signal distortion due to quantization error. c) Plots of the trigger‐in signal, trigger‐out signal, and the current output, into and from an OSC1Lite channel. Fifty individual traces are shown in grey, overlaid with averaged traces in color. The mean (± SD) delay between the rising edges of a trigger‐in signal and the following trigger‐out signal is 17.25 (± 0.04) µs, and that between the trigger‐in and the current output is 30.8 (± 1.3 µs), *n* = 500. d) Circuit diagram of the 48‐channel system. Forty‐eight transistor‐transistor logic (TTL) pulses generated by an AVR‐based microcontroller board (Arduino Mega 2560) trigger the current output. A voltage divider combines 48 TTLs into 4 analog signals which then are fed into analog input channels of the electrophysiology recording system. High‐density and high‐channel count electrodes and μLEDs combined with independent control of any μLEDs enable e) multilayer optotagging, f) neural sequence generation, and g) closed loop optogenetic experiments. e) Schematic of cortical and hippocampal circuitry (blue triangles, green and purple circles represent pyramidal cells, parvalbumin+ and somatostatin+ interneurons, respectively). Note that hectoSTAR optoelectrode can record from and stimulate neurons in cortex and pyramidal layer of CA1 simultaneously. Optoelectrode shank schematic (middle) and stimulation pattern (right) used in head‐fixed mice experiments. 12 μLEDs/shank were used in this stimulation sequence (50 ms stimulation interleaved with 100 ms no stimulation). f) OSC1Lite‐controlled time‐varying neural sequences can be generated in CA3 and the resulting activity can be recorded along the CA1‐CA3 axis of the hippocampus. g) Behavioral or neural events can trigger OSC1Lite which in turn can deliver current to any μLED within 35 µs. Depending on the animal position OSC1Lite can activate μLEDs while a mouse is running on a track (location of stimulation is shown by infrared LEDs).

An open‐source custom‐built multi‐channel μLED controller system with a graphical user interface was designed to enable the independent control of multiple μLEDs on the hectoSTAR μLED optoelectrode (see Section 4). At the core of the system is a 12‐channel FPGA‐based optical stimulation controller (Optical Stimulation Chip Version 1‐Light, OSC1Lite; **Figure** **3**a and Figure S5, Supporting Information). OSC1Lite was designed to allow independent manipulation of current output from multiple channels in a closed‐loop setting. In addition, arbitrary waveforms can be generated from the output of each channel so that a variety of stimulation profiles can be generated (Figure S6, Supporting Information). The current output from each channel is updated every 17.2 µs at 1 µA resolution so that arbitrary current signal can be generated at a high fidelity (Figure 3b). OSC1Lite responds to trigger‐in pulses by immediately sending out a trigger‐out pulse within 17.25 ± 0.04 µs (Figure 3c; mean ± SD, *n* = 500) and generating the current signal within 30.8 ± 1.3 µs (Figure 3c; mean ± SD, *n* = 500, measured at 50% transition points). An AVR‐based microcontroller board (Arduino Mega2560, Arduino, Italy) was utilized to generate trigger signals for 48 individual channels (Figure 3d). As shown in Figure 3d, the trigger‐out pulses generated from OSC1Lite channels were multiplexed into 4 analog signals and then fed into the electrophysiology signal recording system (RHD USB Interface Board, Intan Technologies), so that the accurate timestamps of the optical stimulation can be recorded by the recording system and synchronized with electrophysiology recordings.

Composed of four custom‐built μLED controllers connected in parallel, the system allowed real‐time control of up to 48 independent LEDs on a μLED optoelectrode during in vivo experiments. The system was built with commercially available off‐the‐shelf circuit components and therefore provides an effective yet economical way to utilize the high‐precision optical stimulation capability of hectoSTAR μLED optoelectrodes.

The flexibility of the patterns that can be generated from each channel of the controller system combined with the short latency between the trigger‐in signal and current generation allows the utilization of the system in multiple experimental designs (Figure S7, Supporting Information). Figure 3e‐g illustrates three examples of unique experiments enabled by this technology. HectoSTAR μLED probes allow simultaneous optogenetic tagging of genetically defined cell types across layers and structures and the study of their functional interactions (Figure 3e). To understand how upstream inputs are read out by downstream target structures, artificial input patterns can be generated in the former while recording both neural populations (Figure 3f). Finally, the short latency of the controller circuit even allows closed loop optogenetics experiments to be performed, with stimulation triggered by either behavioral or neural events (Figure 3g).

### 2.4 Investigation of Inter‐Areal Cell Type‐Specific Interactions

**Figure 4:**
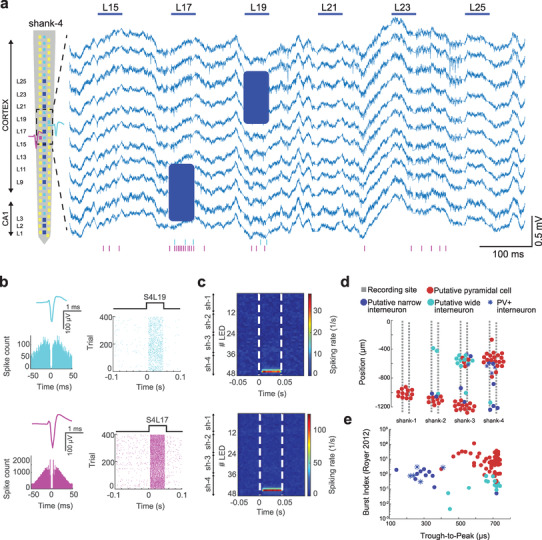
Multi‐regional recording in a head‐fixed mouse using hectoSTAR μLED optoelectrode. a) LFPs recorded on 11 channels in a PV::ChR2 mouse. Left: single shank layout shows the location of activated μLEDs used in this experiment (dark blue). 50 ms light pulses delivered by LED‐15‐25 (horizontal blue lines) induced spiking activity of single units (blue shaded area). Raster plot at the bottom shows the activity of two single units during μLED stimulation. Putative position of the example neurons relative to the recording sites is shown on the left. b) Mean waveforms and autocorrelation histograms indicate well isolated single units. On the right, single LED‐triggered raster plots are shown for the two cells. Cell in cyan was significantly modulated by shank‐4 LED‐19 (*p* < 0.01, bootstrap test), and the cell in magenta was modulated by shank‐4 LED‐17 (*p* < 0.01). c) LED triggered mean spiking rate is shown for each cell. Each row represents the average spiking rate of the neuron triggered by an LED (*n* = 396 trials, LED1‐12 is on shank‐1, LED13‐24 is on shank‐2, LED25‐36 is on shank‐3 and LED37‐48 is on shank‐4, white dashed lines show the onset and offset of light stimulus). The cells are significantly modulated by the following LEDs (cyan cell: LED43‐45, spiking rate: 4 Hz, 14.2 Hz and 26.75 Hz, CI = 0.8–3.1, magenta cell: LED42‐46, spiking rate: 10.64 Hz, 48.44 Hz, 105.64 Hz and 15.9 Hz, CI = 4.3–10.4). d) Probe layout is shown with the putative location of recorded neuron somata (*n* = 61 putative pyramidal cells, 15 narrow interneurons and 12 wide interneurons). Single units were clustered in the cellular layers of cortex and hippocampus (0 µm represents brain surface). e) Clustering of neurons by through‐to‐peak time of their waveform and burst index. Note the separation of narrow waveform interneurons and pyramidal cells. Blue stars indicate the optotagged PV+ cells recorded during the same session as in (a).

To demonstrate the capabilities of the hectoSTAR μLED optoelectrode for in vivo interrogation of neural circuits, we performed acute recordings in head‐fixed mice (Figure S8, Supporting Information). The hectoSTAR probe was inserted in the dorsal hippocampus targeting cortex and CA1 simultaneously (**Figure** **4**a,d). Some of these experiments also targeted dorsal CA1, CA3 and dentate gyrus subregions (Figures S9a and S10, Supporting Information). Laminar LFP recordings allowed the identification of cellular and dendritic layers based on electrophysiological markers. In addition to wide‐band LFPs, low impedance electrodes enable high signal‐to‐noise recordings of extracellular spikes, with more than 200 µV in many cases (Figure 4a,b and Figure S1, Supporting Information). Spikes from individual neurons were recorded from multiple electrodes simultaneously (3–5 electrodes typically) due to their high‐density and staggered arrangement in the probe shank. After semi‐automatic clustering of recorded spikes, >700 single units were isolated across nine recording sessions (84 ± 28 single units/session, mean ± SD; *n* = 7 mice). These units were classified into putative cell types based on waveform and spike train characteristics (Figure 4b,e, criteria of this classification can be found in Section 4). Physiological classification of cell types is prone to errors and only allows a coarse division into broad categories such as excitatory or inhibitory cells. To further refine such classification and provide ground truth data, we performed optogenetic “tagging” of genetically defined cell types by delivering brief pulses of light through individual μLEDs in different transgenic mice lines expressing ChR2 selectively in parvalbumin expressing (PV+) or somatostatin expressing (SOM+) inhibitory cells or CamKII expressing excitatory cells (CamKII+). 50 ms light pulses delivered by individual μLED elicited reliable discharges of action potentials with short latency in nearby cells expressing ChR2 (Figure 4b,c and Figure S9, Supporting Information). Illumination of μLEDs on neighboring shanks did not induce time‐locked spiking of neurons (Figure 4c). This approach provided ground truth data to guide the classification of these three cell types in our recordings (Figure 4d,e).

The capability of multi‐region recording and identification of genetically defined cell types with the hectoSTAR optoelectrode enabled a novel approach to study circuit interactions in behaving animals. We focused on the circuit integrated by the hippocampal CA1 area and its main input region, the CA3 area. We first identified putative monosynaptically connected pyramidal—interneuron cell pairs as determined by the cross‐correlograms of their spike trains. The presence of a significant short‐latency (1–3 ms) peak in the cross‐correlogram denoted a functional monosynaptic cell pair (Figure S11a, Supporting Information).^[^ ^43^ ^]^ We found multiple examples of such functionally connected cell pairs across hippocampal subregions, including from optogenetically tagged CamKII+, PV+ and SOM+ cells (Figure S11b, Supporting Information). Taking advantage of the 2D recording with the hectoSTAR optoelectrode, we characterized the spatial distribution of monosynaptic interaction motifs in these cell types. The number and strength of pyramidal‐interneuron monosynaptic connections followed a log‐normal distribution, with most cells having few and weak connections and a minority having a large number of connected pairs (up to 14). We found that PV+ had on average more connections per cell than SOM+ or CamKII+ cells (3.53 ± 2.63 connections per cell for PV+, 2 ± 1.41 for SOM+, and 2.13 ± 1.42 for CamKII+). The strength of these connections (spike transmission probability) decayed as a function of the distance between the two cell somas for all cell types (*r* = −0.14 correlation between spike transmission probability and distance for *n* = 220 pairs, *p* < 0.05, rank‐sum test).

**Figure 5:**
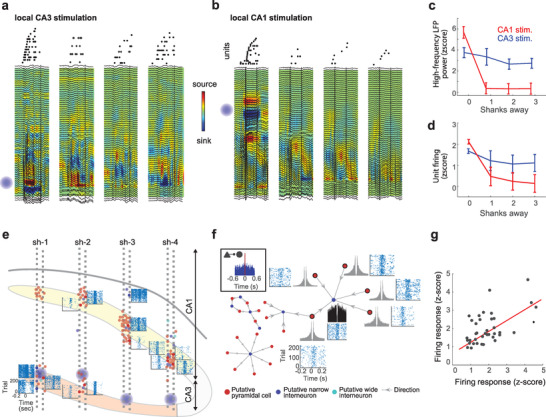
Unveiling cell type‐specific interactions in the CA3‐CA1 circuit. a) Example response elicited by a 100 ms single μLED activation in the CA3 (blue globe in shank 1). Top: raster plot of spikes shows sequential activation of cells recorded by each shank. Each dot is one spike and each line an individual neuron. Bottom: depth profiles of LFPs superimposed on current source density (CSD) color maps. Note that oscillatory responses as well as unit firing was elicited in the four shanks. b) Same plot as in (a) but in this case the activated μLED was in CA1 (top on shank 1). Strong LFP and unit response was elicited only in shank 1. c) High‐frequency LFP power (80–200 Hz) in CA1 as a function of the horizontal distance from the activated μLED was higher for CA3 than CA1 stimulation (*p* < 0.001, rank‐sum test, for sites 1–3 shanks away). d) Unit firing response in CA1 was also stronger for CA3 than CA1 simulation for sites 1–3 shanks away of the activated μLED (*p* < 0.01, rank‐sum test). e) Location of recorded neuron somatas (*n* = 89 pyramidal cells, 19 narrow‐waveform interneurons and 3 wide‐waveform interneurons, red, dark blue and light blue, respectively) imposed on probe layout (CA1 and CA3 are shown in yellow and orange, respectively). Raster plots show single cell responses to 120 ms light pulses delivered by individual μLEDs in CA3 (blue globes). f) Top left inset: Example functional monosynaptic connection identified from the CCG between a putative pyramidal cell and interneuron. Different functional connectivity motifs from the same session are illustrated with directed graphs (arrows indicate the direction of connection between neurons). Autocorrelation histograms and CA3 optogenetic sequence triggered raster plots are shown for each highlighted neuron. g) Correlation between the firing rate elicited by CA3 stimulation of CA1 cells with mutual monosynaptic connections (*r* = 0.51, *p* = 0.0008; *n* = 50 pairs).

We then asked a question that was difficult to tackle with conventional optogenetic tools: Is CA3 to CA1 in vivo functional connectivity related to downstream cell types and local connectivity? Short pulse stimulation (120 ms) with a single μLED located in the CA3 pyramidal layer elicited a strong response in both CA3 and CA1 regions in the four shanks (a spread of activity of >1 mm) (**Figure** **5**a–d). However, the same stimulation delivered with single μLEDs located in the CA1 pyramidal layer elicited a strong response but only locally (Figure 5b–d), likely due to the lack of strong recurrent excitatory connections as in CA3. We found responses of different cell types in both CA1 and CA3 to CA3 local stimulation (Figure 5e). Response latencies were longer for CA1 than CA3 cells (*p* = 0.036, rank‐sum test). CA1 PV+ cells were more likely to discharge in response to local CA3 stimulation than CA1 CamKII+ or SOM+ cells (52/5/27% of responsive PV+/SOM+/CamKII+ cells) and did so with shorter latencies (*p* < 0.05, rank‐sum test).

In each recording session we found multiple motifs of local functional connectivity in CA1, that is, several interneurons connected to the same pre‐synaptic pyramidal cell or vice versa (Figure 5f and Figure S11, Supporting Information). We thus analyzed if downstream connectivity (in CA1) was related to upstream inputs (from CA3). Indeed, we found that the magnitude of response to CA3 stimulation was significantly correlated for CA1 cells that had local monosynaptic connections (*r* = 0.51, *p* < 0.001) but not for un‐connected cells (*r* = ‐0.014, *p* > 0.05), revealing the existence of inter‐regional functional connectivity motifs (Figure 5g).

### 2.5 Dissection of Input–Output Transformation across Brain Regions

The CA3/CA2 area generates synchronous network patterns known as sharp‐wave ripples (SPW‐Rs) that propagate to CA1 eliciting a strong activation of local cell ensembles.^[^ ^4^, ^44^ ^]^ The sequential order in which CA1 cells fire during SPW‐Rs recapitulates recent experience, and it has been suggested that it constituted a cellular mechanism for memory consolidation and action planning.^[^ ^4^ ^]^ Two main patterns of activity during SPW‐Rs have been described. Hippocampal cells can reactivate in the same order that they fired during behavior (“forward sequences”) or in reverse order (“reverse sequences”). Different functional roles have been attributed to either type of pattern,^[^ ^45^, ^46^ ^]^ but their underlying mechanisms remain unknown. A pre‐requisite for such neuronal sequences role in memory is that downstream regions can effectively distinguish among them.

**Figure 6:**
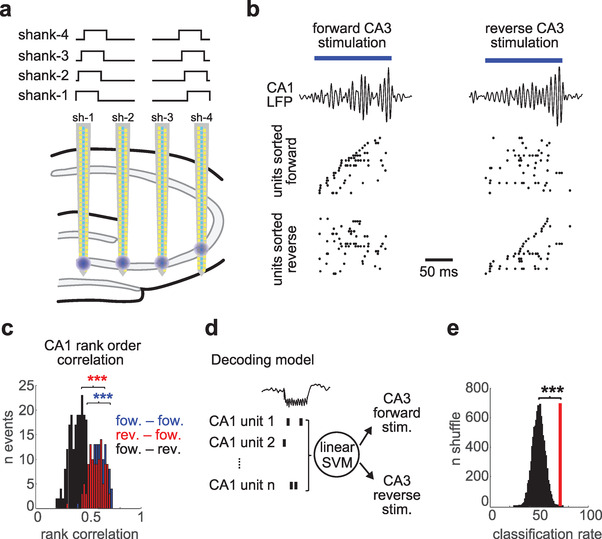
Readout of input patterns from upstream population activity. a) Experimental design. Individual μLEDs from each shank (bottom; blue globes) were sequentially activated in a forward or reverse manner (top) to stimulate CA3 neurons. b) Example response in CA1 to forward (left) and reverse (right) CA3 stimulation. CA1 filtered LFP (80–300 Hz) is shown on top and unit population response below (only CA1 pyramidal cells that fired during these events were included). In the first column units were sorted according to their firing order during forward CA3 stimulation, and on the second column by their order during reverse stimulation. c) Rank order correlation for CA1 sequences during forward and reverse CA3 stimulation events (*n* = 120/120 forward and reverse events) ***p < 0.001, rank‐sum test. d) Schematic of decoding approach. Spike trains of CA1 pyramidal cells during CA3 stimulation events were used as input features for the linear SVM to decode input pattern (either forward or reverse stimulation). e) Decoding accuracy compared to shuffle distribution. Red line indicates decoding accuracy (71.4%) and black histogram shuffle distribution (*p* = 0.0004; 10000 shuffles).

We took advantage of our simultaneous CA1–CA3 recordings to test this hypothesis. We delivered two patterns of CA3 stimulation in an interleaved manner by sequentially activating μLEDs located in the CA3 pyramidal layer of each shank in either a forward or reverse sequence (120 ms partially overlapping pulses, **Figure** **6**a). This experiment was conducted in CamKII::ChR2 transgenic mice (*n* = 3), so only pyramidal cells were directly activated. Such stimulation entrained local CA3 cells and induced a SPW‐R in upstream CA1 (Figure 6b). CA1 neurons were activated in a different sequential pattern during forward and reverse CA3 stimulation (Figure 6c).

To quantify this phenomenon, we analyzed the rank‐order correlation of CA1 activity during stimulation events.^[^ ^45^ ^]^ CA1 sequences in response to the same type of CA3 stimulation were more correlated than when forward versus reverse events were compared (Figure 6c). To directly test if CA1 ensembles could distinguish between forward and reverse input sequences, we employed a Support Vector Machine (SVM) decoding approach. We used SVM to perform a binary linear classification of CA1 population responses to CA3 forward and reverse stimulation patterns (Figure 6d). We took the spike trains of CA1 neurons during the 120 ms of CA3 stimulation and used them as input features to the SVM decoder. Half of the data was used to train the decoder (*n* = 120 events) and decoding accuracy was tested on the remaining half. We found that our decoder predicted the correct CA3 input pattern 71.4% of the time, which was highly significant compared to a shuffle distribution where CA3 input labels were randomly assigned (Figure 6e; *p* = 0.0004; bootstrap test). These results suggest that CA1 can effectively readout the sequential order of its CA3 inputs.

## 3 Discussion

We have presented here the fabrication and testing of the hectoSTAR μLED optoelectrode. This silicon‐based probe features 256 electrodes and 128 LEDs, distributed on four shanks and covering a large volume with a cross‐sectional area of 900 µm × 1300 µm. The highest packing density of light sources and the electrodes combined to date, to the best of our knowledge, was achieved with the integration of an order of magnitude more electrodes and μLEDs than on any other previously reported optoelectrodes. In addition, we developed a micro‐controller for independent control of μLEDs to deliver stimulation light with arbitrary patterns. We demonstrated the unique capabilities of the device for high‐resolution selective neuronal modulation and recording in behaving mice. Previous optoelectrodes also enabled the recording and stimulation of neurons with high spatio‐temporal resolution but they spanned a very limited volume of tissue (≈250 × 800 µm) (Table S4, Supporting Information). These reduced dimensions made it impossible to simultaneously record with one of these devices more than one brain area. Although it would be theoretically possible to record with more than one of those devices in the same animal, such experiments have not been conducted so far due to their technical difficulty, even more when the regions of interest are very closely located, as it is the case of the CA1 and CA3 hippocampal areas. The main feature of the hectoSTAR optoelectrode is the ability to perform multi‐region (such as neocortex and hippocampus, or CA1 and CA3 hippocampal subregions) high‐density recordings of neuronal ensembles together with optogenetic stimulation with near single‐cell resolution at scale. This allows us to dissect network interactions of defined cell types across hippocampal sub‐regions.

The hectoSTAR optoelectrode is particularly suitable to address one of the main goals in systems neuroscience – to understand input‐output transformations in neural circuits. To date, such inference is typically done by simultaneously recording connected regions and correlating the patterns of activity across regions. While such correlational approaches have provided valuable insights on the mechanism of neural communication, perturbation methods are needed to test the conclusions based on correlations. The hectoSTAR optoelectrode enables one to investigate in vivo the properties of CA3 to CA1 functional inputs. First, we demonstrated that low‐intensity local stimulation with an individual μLED (likely only directly depolarizing a few pyramidal neurons in the immediate vicinity of a given μLED) can synaptically entrain their partner interneurons in both CA1 and CA3 regions. Different types of postsynaptic CA1 cells were activated by optogenetically driven CA3 inputs and showed different response properties. PV+ cells (a group that includes subsets of basket, bistratified, and axo‐axonic interneurons)^[^ ^47^ ^]^ were more likely to be activated by CA3 inputs than SOM+ or CamKII+ cells and responded with shorter latencies (Figure 5). This confirms previous observations of a strong feedforward inhibitory component of the CA3 to CA1 input and suggests that it is mediated by PV+ interneurons.^[^ ^48^ ^]^ Furthermore, we also found that the local motifs of connection in CA1 influence the response to extrinsic CA3 inputs. CA1 cells that shared functional monosynaptic connections were more strongly correlated in response to CA3 stimulation, compared to other, non‐connected CA1 cells (Figure 5). This result suggests the existence of inter‐areal functional connectivity motifs. Further research is necessary to investigate the origin and functional role of such motifs in CA3 to CA1 communication.

The second novel observation enabled by the hectoSTAR optoelectrode was that CA1 neuronal ensembles can reliably read out the sequential order of their CA3 inputs (Figure 6). This is an important finding because SPW‐R‐associated sequences have been postulated as the cellular hallmark of learning and memory in the hippocampus.^[^ ^4^, ^49^ ^]^ The order in which hippocampal cells fire during SPW‐R recapitulates recent experience, a phenomenon termed “replay”.^[^ ^50^, ^51^ ^]^ Replay can proceed in the same or opposite order as the neurons that were active during behavior (“forward replay” or in the opposite order “reverse replay”). Numerous studies have suggested that the content of replay, i.e., the order of activation of neuronal ensembles, is fundamental for learning and memory.^[^ ^45^, ^46^, ^52^ ^]^ A pre‐requisite for this hypothesis is that these sequences need to be read out by downstream target regions. CA1 neuronal population could distinguish between the forward and reverse order of activation of their upstream CA3 pyramidal cells (Figure 6). In these experiments, the same individual cells were activated in response to both types of inputs, the main difference was only the order in which they fired. Overall, these results provide support for the hypothesis that neuronal sequences are an effective code of communication between brain regions.

The utility of the hectoSTAR μLED optoelectrodes may further be improved with additional engineering innovations. A key step is the miniaturization of the back end of the device to enable experiments in freely moving mice and other small animals. Here, we demonstrated the capabilities of the hectoSTAR μLED optoelectrode in head‐fixed animals. The size of an unpackaged device is quite small, measuring 4.2 × 11.1 × 0.03 mm (*W* × *L* × *T*), including the backend for the external connection. The printed circuit board, on which the connectors for the interface with the recording and the stimulation system are integrated in the current instantiation is large and prevents its practical use in freely moving small rodents. It is expected that, if miniature‐sized interface circuit(s) with wire bonding pads in appropriate dimensions and layouts can be integrated with the circuit(s) by the means of a flexible cable (i.e., using microflex technology),^[^ ^53^ ^]^ the size of the packaged device can be reduced enough so that it can be mounted on the head of a freely moving mouse.

Previous work has demonstrated the utility of flexible electrode interfaces to record the same neurons for extended periods of time.^[^ ^6^, ^54^, ^55^, ^56^ ^]^ A caveat of our approach is the rigidity of the silicon substrate employed in the fabrication of the probe. Thus, a promising future extension of this work will be the development of version of the hectoSTAR μLED optoelectrode in a flexible (e.g., paralyne) instead of rigid silicon substrate. Towards that goal, a number of engineering challenges remain to be overcome, including the monolithic integration of multi‐color LEDs on a silicon substrate, the high‐yield transfer of μLEDs onto a flexible substrate, and a defect‐free yet flexible polymer encapsulation. Techniques for the monolithic integration of multi‐color LEDs^[^ ^57^, ^58^, ^59^ ^]^ and those for the wafer‐level transfer of μLED from a rigid substrate to a flexible substrate^[^ ^60^, ^61^, ^62^ ^]^ have been recently demonstrated by several research groups. We anticipate that these techniques will come to maturity in the near future and be incorporated for the fabrication of next‐generation hectoSTAR μLED optoelectrode.

Another promising avenue of future development is the integration of nanoscale electrodes that enable intracellular electrophysiological recording^[^ ^1^, ^2^ ^]^ onto the hectoSTAR μLED optoelectrode. Such intracellular recording capability would certainly provide a great advantage of being able to monitor important sub‐threshold neuronal activity, to which extracellular recordings do not provide access. At the same time, one must carefully evaluate the intrinsic limitations of intracellular recordings: typical short duration recordings (< 30 min) due to membrane damage (and thus cell death), low cell yield, etc. For experiments that require a continuous monitoring of large neuronal populations over a course of extended time, the use of intracellular electrodes will be sub‐optimal; however, its combination with simultaneous large‐scale extracellular recordings and optogenetic manipulations could offer an invaluable tool for the dissection of neural circuit mechanisms.

## 4 Experimental Section

### HectoSTAR μLED Optoelectrode Fabrication and Packaging

All the microfabrication steps were carried out in Lurie Nanofabrication Facility, University of Michigan, Ann Arbor, MI, USA. The procedure for the fabrication of μLED optoelectrodes in a multi‐metal‐layer configuration^[^ ^33^ ^]^ was utilized, and a fine‐pitch photolithography technique was employed to define narrow metal lines, serving as LED and electrode interconnects, on the surface of optoelectrode. More specifically, 100‐nm thick and 700‐nm wide gold (Au) lines were defined by lift‐off patterning process utilizing a thin bilayer resist and a 5× image reduction i‐line step‐and‐repeat projection photolithography tool (GCA AutoStep 200). A thin layer of contrast enhancing material was spin‐coated on top of the resist stack just before the exposure and was immediately rinsed away. For each metal layer, a 90‐µm thick Au layer was electron beam evaporation deposited (Enerjet, Denton Vacuum, Moorsetown, NJ, USA) on the patterned wafer after a 10‐nm titanium (Ti) as the adhesion layer. Dissolution of the bilayer resists in a warm (40 °C, overnight soak) bath of *N*‐Methyl‐2‐pyrrolidone(NMP)‐based solvent (Remover PG, Kayaku Advanced Materials) completed the lift‐off process.

Fabricated hectoSTAR optoelectrodes were packaged on printed circuit boards that provide interface to external signal conditioning electronics. Two‐layer printed circuit board with 2‐mil (0.051 mm) half‐pitch, 0.7‐mil thick (half ounce, 0.018 mm) copper traces were fabricated at a commercial PCB fabrication facility (Hughes Circuits, San Marcos, CA, USA). Electrical connectors for the connection of the PCB with neuronal signal recording headstage (Molex SlimStack 502430–6410, Molex LLC, Lisle, IL, USA) and LED drivers (NPD‐36‐AA‐GS, Omnetics Connectors Corp., Minneapolis, MN, USA) were reflow soldered on the PCB before attaching the optoelectrode to the printed circuit board.

A separately fabricated, 4‐µm thick polyimide flexible interposers^[^ ^53^ ^]^ were utilized to provide electrical and mechanical connections between the printed circuit board and the optoelectrode. The polyimide cables containing embedded metal lines was fabricated on a silicon wafer and then released from the wafer. Gold ball bumps were formed on both ends of the metal lines to form vertical connections from the metal lines on the cable to the pads located underneath. A ball bonder (K&S 4524‐D, Kulicke and Soffa Industries, Inc., Fort Washington, PA, USA) was utilized for the formation of ball bumps. After all the components were attached to the printed circuit board, all the exposed metallic surfaces were covered with thermal epoxy (EPO‐TEK 353ND and 353ND‐T, Epoxy Technologies, Billerica, MA, USA) for protection.

### Electrical and Optical Characterization of hectoSTAR μLED Optoelectrode

The current–voltage (*I* vs *V*) and the radiant flux‐current (*Φ*_e_ vs *I*) characteristics of each μLED on the packaged hectoSTAR μLED optoelectrodes were measured. A source meter (Keithley 2400, Keithley Instruments, Cleveland, OH) was utilized to provide voltage across the anode and the cathode of a μLED on the optoelectrode, and a multiplexer (Keysight 34908A on 34970A, Keysight Technologies, Santa Rosa, CA, USA) was placed between the source meter and the optoelectrode to provide the automatic channel multiplexing capability. An optical measurement system consisting of an integrating sphere (FOIS‐1, Ocean Optics, Largo, FL, USA) and a spectrometer (Flame, Ocean Optics) was utilized for the optical measurement. First, the tips of the optoelectrode were moved until the shanks were completely inside the integrating sphere, ensuring that all the light generated from the μLED can be collected. The DC voltage across the terminals of each LED was swept from 0  to 6.5 V with 75 µA current compliance, and the current output from the source and the spectral flux the spectrometer detected were recorded. The radiant flux was calculated by integrating the spectral flux over wavelengths from 350 to 600 nm.

The 1‐kHz impedance of each recording electrode on the hectoSTAR μLED optoelectrode was measured using an Intan neural signal recording headstage (RHD 128‐channel headstage, Intan Technologies, Los Angeles, CA, USA) inside 1 × phosphate‐buffered saline (PBS) solution (prepared using 10 × PBS purchased from MP Biomedicals, Solon, OH, USA). Impedances of the electrodes were measured using a neuronal signal recording system (RHD2000, Intan Technologies, with RHD2000 interface software v 1.5.2), 128 channels at a time. First, a 250 mL beaker was filled with 1 × PBS. After connecting the headstage to a pair of Molex connectors on the optoelectrode PCB, the μLED optoelectrode was lowered into the container until the bottom halves of the shanks (∼ 3 mm) were submerged in the PBS. Exposed tips of the reference wires, whose other ends are soldered to the vias of the corresponding pins on the headstage, were also submerged in the PBS. After measuring impedance of the first 128 electrodes using the automatic impedance measurement feature of the Intan software, the headstage was moved to the other pair of the Molex connectors and the impedance of the rest of the electrodes were measured.

### Simulations for hectoSTAR μLED Optoelectrode Performance

The attenuation (insertion loss, IL) and the crosstalk (far‐end crosstalk, FEXT) of recording electrode signals were simulated for a hectoSTAR μLED optoelectrode. First, an equivalent 3D model was built for a 100‐µm portion of an optoelectrode shank (Figure S2a, Supporting Information) using COMSOL (COMSOL Multiphysics 5.4, COMSOL Inc., Burlington, MA, USA). The capacitance values in the equivalent circuit model (Figure S2b, Supporting Information) were calculated using a stationary electrostatics analysis, and the resistance values were calculated from the measured sheet resistivities of the corresponding metal layers. Then, a netlist of a circuit, consisting of a T‐network (unit cell shown in Figure S2c, Supporting Information) and the other components in the signal recording circuit, was built and simulated using LTSpice (LTSpice XVII, Analog Devices, Wilmington, MA, USA). The magnitudes and the phases of the voltage signals recorded at the target node (*V*_o1_) and the neighboring node (*V*_o2_) were calculated *V*_i1_ = 1 V, for the 1 mHz–1 MHz frequency band. The values of the capacitance and the resistances are shown in Table S1, Supporting Information.

Light intensity distribution in the brain tissue was simulated using a model of a brain tissue and a planar 8 µm × 11 µm light source representing a μLED. A Helmholtz equation describing the light fluence rate inside a turbid medium with large scattering and absorption coefficients^[^ ^63^, ^64^ ^]^ was solved using COMSOL, with the surface of the optoelectrode modeled as an ideal reflective surface. Absorption coefficient (*μ*_a_) and reduced scattering coefficient (µs') utilized were 4.47 and 50.5 cm^–1^, respectively.^[^ ^64^ ^]^

Tissue heating was simulated using a model of a brain tissue (7 mm × 7 mm × 7 mm, *W* × *H* × *L*) and a 6‐mm long silicon needle implanted inside the tissue. Heat equivalent to the amount of electric power provided to the LED (300 µW) was assumed to be generated from an 8 µm × 17 µm × 0.5 µm (*W* × *L* × *H*) volume, which corresponds to the location of the lowermost μLED. The silicon needle was built with dimensions identical to those of a shank of the hectoSTAR optoelectrode, and a 1‐µm thick silicon dioxide layer was assumed to be covering the top surface of the 30‐µm‐thick silicon shank. All metal traces and the GaN/InGaN LED stack were ignored for simplicity, as their thicknesses are negligible and their thermal properties comparable to those of silicon. Pennes’ bioheat transfer equation^[^ ^65^ ^]^ was solved using COMSOL, with the top surfaces and the exposed sides of the silicon needle assumed to be thermally insulating and the rest of the surfaces assumed to be isothermal. The thermal properties of the brain tissue and the implanted optoelectrode are provided along with the coefficients of bioheat transfer equation in Table S2, Supporting Information.

### Acute Animal Experiments

The animal procedure was approved by the Institution Animal Care and Use Committee of the University of Michigan (protocol number PRO‐7275). One male transgenic mouse (JAX stock #0 07612) was utilized for the experiment. Electrophysiology recordings were made using two RHD 128‐channel recording headstages (Intan technologies, Los Angeles, CA) connected to the PCB on which the probe was mounted via two pairs of Molex SlimStack (502426‐6410, Molex, Lisle, IL) connectors. A PC running Intan data acquisition software, connected to an Intan USB interface board via a USB 2.0 cable, was utilized to acquire and save data in real‐time. NeuroScope^[^ ^66^ ^]^ was utilized for the real‐time visualization of data collected from all the 256 channels. Voltage signals for the LED driving were provided using a function generator (33220A, Keysight Technologies, Santa Rosa, CA). Rectangular voltage pulses with 0 V low‐level voltage and 3.5 V high‐level voltage were used as the driving signal, and one‐hundred‐millisecond‐long pulses were applied every 5 s in cortex and hippocampus (*n* = 260 pulses, Figures S12 and Figure S13, Supporting Information).

### Head‐Fixed Animal Experiments

All experiments were approved by the Institutional Animal Care and Use Committee at New York University Medical Center. Animals were handled daily and accommodated to the experimenter before the surgery and head‐fixed recording. Mice (adult male *n* = 4 CaMKII‐ChR2, *n* = 2 PV‐ChR2, and *n* = 1 somatostatin‐ChR2 mice, 26–31 g) were kept in a vivarium on a 12‐h light/dark cycle and were housed two per cage before surgery and individually after it. Atropine (0.05 mg kg^−1^, s.c.) was administered after isoflurane anesthesia induction to reduce saliva production. The body temperature was monitored and kept constant at 36–37 °C with a DC temperature controller (TCAT‐LV; Physitemp, Clifton, NJ, USA). Stages of anesthesia were maintained by confirming the lack of a nociceptive reflex. The skin of the head was shaved, and the surface of the skull was cleaned by hydrogen peroxide (2%). A custom 3D‐printed headpost^[^ ^67^ ^]^ (Form2 printer, FormLabs, Sommerville, MA) was attached to the skull using C&B Metabond dental cement (Parkell, Edgewood, NY). The location of the craniotomy was marked and a stainless‐steel ground screw with header pin was placed above the cerebellum. Each animal recovered for at least 7 days prior to habituation of the head‐fixation. Animals were allowed to walk freely on a low‐friction rodent‐driven belt treadmill during recording sessions (Figure S8, Supporting Information).^[^ ^68^ ^]^ The day before recording, a craniotomy was performed (2 mm posterior from Bregma and 1.5 mm lateral to midline) and the dura was removed. After the surgery, the craniotomy was sealed with Kwik‐Sil (World Precision Instruments, Sarasota, FL) until the recording. On the day of the recording the animal was head‐fixed, the craniotomy was cleaned and the headpost was filled with sterile saline. The ground of the probe PCB was connected to the header pin and the probe was inserted to the target depth using a manual micromanipulator (MM‐33, Sutter Instruments, Novato, CA). The authors constantly monitored the electrophysiological signal during insertion. The collected data was digitized at 20 kS s^−1^ using an RHD2000 recording system (Intan technologies, Los Angeles, CA). The authors waited at least 15 min after reaching the target depth. Baseline session and optogenetic stimulation session(s) were recorded from each mouse. After the recording session, the craniotomy was sealed with Kwik‐Sil, and the animal was put back into its homecage. If more than one session was recorded from an animal, a new craniotomy was prepared as described above on the contralateral side.

### μLED Control

Current‐controlled stimulation was used to drive individual μLEDs (OSC1Lite, 12‐ch current source (https://github.com/YoonGroupUmich/osc1lite). Each shank was controlled by an OSC1Lite. The location of the activated μLEDs were selected before the experiment and remained the same throughout a recording session. The stimulation amplitude and waveform were defined using OSC1Lite's open‐source graphical user interface. Current was delivered to the individual μLEDs using a 36‐pin Omnetics cable (A79029‐001) attached to a solderless breadboard (Figure S6, Supporting Information). 48 digital outputs of an Arduino Mega 2560 microcontroller were connected to the trigger input of 4 OSC1Lites. Predefined stimulation sequences were uploaded to the Arduino board before the experiments and a manual switch triggered the sequence. The Arduino code was running until the manual switch was turned off. All stimulation parameters are listed in Table S3, Supporting Information.

### Local Field Potential Analysis

Ripple detection and wavelet spectrogram calculation were performed as previously described.^[^ ^44^, ^69^ ^]^ To detect ripples a single electrode in the middle of the pyramidal layer was selected. The wide‐band LFP signal was band‐pass filtered (difference‐of‐Gaussians; zero‐lag, linear phase FIR), and instantaneous power was computed by clipping at 4 SD, rectified and low‐pass filtered. The low‐pass filter cut‐off was at a frequency corresponding to p cycles of the mean band‐pass (for 80–250 Hz band‐pass, the low‐pass was 55 Hz). Subsequently, the power of the non‐clipped signal was computed, and all events exceeding 4 SD from the mean were detected. Events were then expanded until the (non‐clipped) power fell below 1 SD; short events (<15 ms) were discarded. To analyze high‐frequency oscillatory activity in the LFP at a high resolution in time and frequency, the complex wavelet transform of the LFP was calculated using complex Morlet wavelets.^[^ ^70^ ^]^ Wavelets were calculated for every 2 Hz frequency step in the 50–150 Hz band. Spectrograms were calculated for each detected SPW‐R or stimulation pulse in a [−150, +150] ms window using the LFP from every individual electrode. Spectrograms for individual events were averaged to construct final plots.

The pyramidal layer of the CA1 region was identified physiologically by increased unit activity and characteristic LFP patterns.^[^ ^44^ ^]^ The identification of dendritic sublayers was achieved by the application of CSD analysis and independent component analysis (ICA) to the LFPs.^[^ ^71^, ^72^ ^]^

### Single Unit Analysis

A concatenated signal file was prepared by merging all recordings from a single animal from a single day. To improve the efficacy of spike sorting, stimulation induced onset and offset artefacts were removed before automatic spike sorting (1ms before and 5 ms after the detected artefacts, linear interpolation between timestamps). Putative single units were first sorted using Kilosort^[^ ^37^ ^]^ and then manually curated using Phy (https://phy‐contrib.readthedocs.io/). After extracting timestamps of each putative single unit activity, peristimulus time histograms and firing rate gains were analyzed using a custom MATLAB (Mathworks, Natick, MA) script.

### Cell Type Classification

In the processing pipeline, cells were classified into three putative cell types: narrow interneurons, wide interneurons, and pyramidal cells. Interneurons were selected by two separate criteria; narrow interneuron is assigned if the waveform trough‐to‐peak latency was <0.425 ms. Wide interneuron was assigned if the waveform trough‐to‐peak latency was >0.425 ms and the rise time of the autocorrelation histogram was >6 ms. The remaining cells were assigned as pyramidal cells.^[^ ^43^, ^69^, ^73^, ^74^, ^75^ ^]^ Autocorrelation histograms were fitted with a triple exponential equation to supplement the classical, waveform feature based single unit classification (https://cellexplorer.org/pipeline/cell‐type‐classification/).^[^ ^76^ ^]^ Bursts were defined as groups of spikes with interspike intervals < 9 ms. The authors had isolated 762 putative single units from seven animals in nine sessions (*n* = 544 putative pyramidal cells, *n* = 152 putative narrow interneurons—10 of them were parvalbumin positive interneurons, and *n* = 66 putative wide interneurons—4 of them are somatostatin positive interneurons).

### Optogenetic Tagging of PV and SOM Cells

To optogenetically tag PV and SOM cells in cortex and hippocampus, PV‐Cre::Ai32 and SOM‐Cre::Ai32 mice were used, respectively. 50 ms light pulses were delivered every 100 ms for at least 200× using 48 μLEDs across four shanks. The spiking activity of each neuron was resampled 500× between the first and last light pulses to build bootstrap samples. Then the authors calculated a bootstrap distribution and confidence interval (0.001–0.999) for each single unit. The putative single unit was categorized as optogenetically activated if the peak spiking rate of the neuron was outside of the bootstrap confidence interval in a 5–8 ms window following light delivery.

### Analysis of Monosynaptic Cell Pairs

Cross‐correlation (CCG) analysis had been applied to detect putative monosynaptic connections.^[^ ^43^, ^77^ ^]^ CCG was calculated as the time resolved distribution of spike transmission probability between a reference spike train and a temporally shifting target spike train. A window interval of [−5, +5] ms with a 1‐ms bin size was used for detecting sharp peaks or troughs, as identifiers of putative monosynaptic connections. Significantly correlated cell pairs were identified using a previously ground‐truth validated convolution method.^[^ ^43^ ^]^ The reference cell of a pair was considered to have an excitatory monosynaptic connection with the referred neuron, if any of its CCG bins within a window of 0.5–3 ms reached above confidence intervals.

### Analysis of CA1 Evoked Sequential Activity

To analyze the evoked population activity in CA1 due to CA3 sequential stimulation, spikes from isolated CA1 pyramidal cells during forward and reverse stimulation periods were collected. Pairwise rank order correlation was calculated either between forward and reverse stimulation events or for each type of events separately. CA1 pyramidal cell unit activity during each stimulation event was transformed into a normalized sequence, using the center of mass of each unit's spikes. Rank distribution of the correlation values was tested against shuffle correlations (1000 shuffles, significance at *p*  =  0.05) using Pearson correlations. To classify CA1 population responses according to the pattern of stimulation in CA3, a linear support vector machine (SVM) classifier was used. The features fed into the classifier were the spike trains of each CA1 unit during stimulation events. The classifier was trained using half of the data in the session and its performance evaluated with the remaining data. Decoding accuracy was computed using a bootstrap test with 10000 surrogate shuffling event labels.

### Statistical Analysis

Statistical analyses were performed with MATLAB functions or custom‐made scripts. No specific analysis was used to estimate minimal population sample or group size, but the number of animals, sessions, and recorded cells were larger or similar to those employed in previous related works.^[^ ^70^, ^72^, ^78^, ^79^, ^80^, ^81^, ^82^ ^]^ The unit of analysis was typically identified as single neurons or assemblies. In a few cases, the unit of analysis was sessions or animals, and this is stated in the text. Unless otherwise noted, non‐parametric two‐tailed Wilcoxon rank‐sum (equivalent to Mann‐Whitney U‐test) or Wilcoxon signed‐rank test was used. For multiple comparisons following ANOVA, Tukey's honesty post‐hoc test was employed. On box plots, the central mark indicates the median, bottom and top edges of the box indicate the 25th and 75th percentiles, respectively, and whiskers extend to the most extreme data points not considered outliers. Outliers are not displayed in some plots but were included in statistical analysis. Due to experimental design constraints, the experimenter was not blind to the manipulation performed during the experiment (i.e., optogenetic manipulation).

## Conflict of Interest

E.Y. and K.D.W. are co‐founders of NeuroLight Technologies, a for‐profit manufacturer of neurotechnology. The remaining authors declare that they have no conflict of interest.

## Supporting information

Supporting Information

Supplemental Movie 1

## Data Availability

The data that support the findings of this study are available from the corresponding author upon reasonable request.
