# Supplementary material for: HectoSTAR μLED Optoelectrodes for Large‐Scale, High‐Precision In Vivo Opto‐Electrophysiology
Source: Adv Sci (Weinh). 2022 Apr 22;9(18):2105414. doi: 10.1002/advs.202105414 (PMC9218760; doi:10.1002/advs.202105414)
Supplement: Supplementary file 1 — Supporting Information [file ADVS-9-2105414-s002.pdf]

Supporting Information

HectoSTAR μLED optoelectrodes for large-scale, high-precision in vivo opto-electrophysiology

Mihály Vöröslakos, Kanghwan Kim, Nathan Slager, Eunah Ko, Sungjin Oh, Saman S. Parizi, Blake Hendrix, John P. Seymour, Kensall D. Wise, György Buzsáki*, Antonio Fernández-Ruiz*, Euisik Yoon*

Supporting Note

Acute tissue damage during insertion

We conjecture that the number of neurons killed per neurons recorded, calculated as the ratio of the volume that the implanted portion of the optoelectrode shanks occupies to the volume from which the electrode can record (a hemispherical volume with a 20-μm diameter), be approximately 5.

OSC1Lite design principles

OSC1Lite hardware employs an FPGA integration module (XEM7001, Opal Kelly Inc., Portland, OR), which is controlled through USB communication between host PC and FPGA (Artix-7, Xilinx Inc., San Jose CA). For a precisely control the optical power output, current based DACs were chosen. Twelve current-based digital-to-analog-converters (DAC8750, Texas Instruments, Dallas TX) are controlled through SPI buses in parallel, and the output of each channel is updated every 17.2 μs. Gain and offset calibration registers on each DAC8750 are adjusted to ensure the actual current output matches the desired value. Additional IC components are integrated on OSC1Lite for reliable operation. First, double capacitive silicon dioxide (SiO2) insulation (ISO7762, Texas Instruments, Dallas, TX) separates the digital signals between the XEM7001 and DAC8750s, eliminating possible surge currents and preventing potential ground loop formation. A series resistor is connected at the output of the DAC to prevent over-current damage to the μLED. A current source (REF200, Texas Instruments, Dallas, TX) is added to draw a constant current of 100 μA from the DAC to compensate for non-zero output offset variations in DAC8750 devices. Two 3.7-V Li-Ion batteries supply power to OSC1Lite, making its power domain separated from the grid.

OSC1Lite operation

An application programming interface (API) and graphical user interface (GUI) provide menu options to select square, trapezoid, or custom current pulses. A user can precisely define the current pulses with a few parameters (period, pulse width, fall and rise times, number of periods to output for one-shot stimulation) and preview the output pulse waveform in the GUI. Channel properties are parameterized (waveform selection, trigger in, trigger out, PC or TTL trigger input, continuous or one-shot stimulation). Prior to an experiment, all the parameters are uploaded in advance to the FPGA. During an experimental session, OSC1Lite

generates pre-defined pulses either at precisely pre-defined timepoints or in response to trigger signals from an external system. The trigger-in mode requires a 3.3-V TTL input for each channel. A 3.3-V TTL trigger-out pulse allows an external system to record the precise timing of the optical simulation.

The FPGA is re-programmable (Verilog), and the software components of OSC1Lite is open source. Therefore, a user is able to add any custom logics and additional controls. For example, a user may implement the program that interprets an additional trigger-in TTL pulse and uses it to halt stimulation.

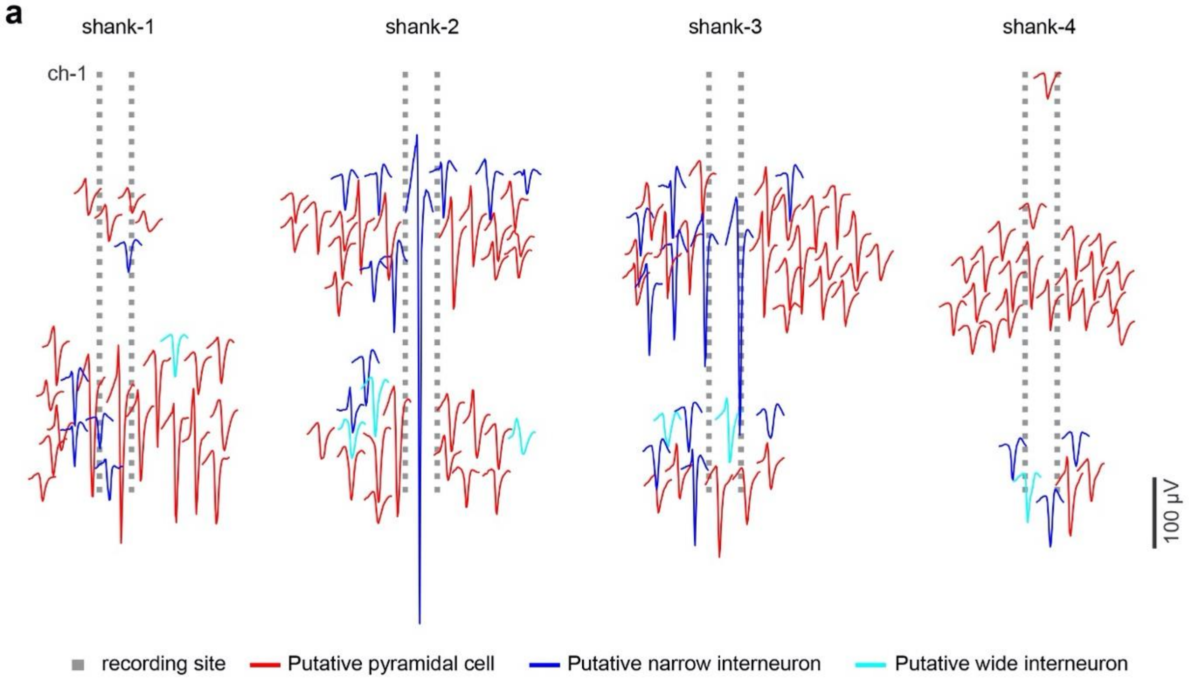

Figure S1. Single unit activity recorded with hectoSTAR µLED optoelectrode. a) Simultaneously recorded well isolated single units recorded with hectoSTAR µLED optoelectrode from the hippocampus of a head fixed CaMKII+ mouse. The mean waveform of each neuron is shown at the location of the maximum waveform amplitude (n = 140 putative single units, red is putative pyramidal cell, blue is putative narrow interneuron and cyan is putative wide interneuron). Channel-1 (ch-1) corresponds to the location of the topmost channel of the shank.

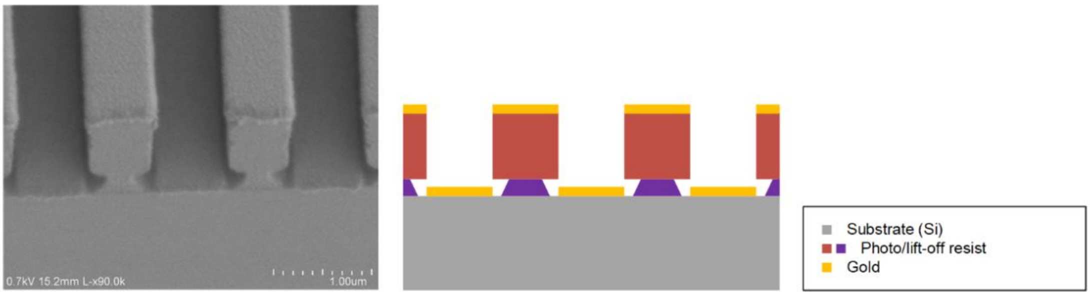

Figure S2. Cross-sectional view of 100-nm thick gold deposited over a patterned sacrificial lift-off resist stack, captured using scanning electron microscopy. A stepped sidewall profile created by the bi-layer resist, which is favorable for lift-off process, can be observed.

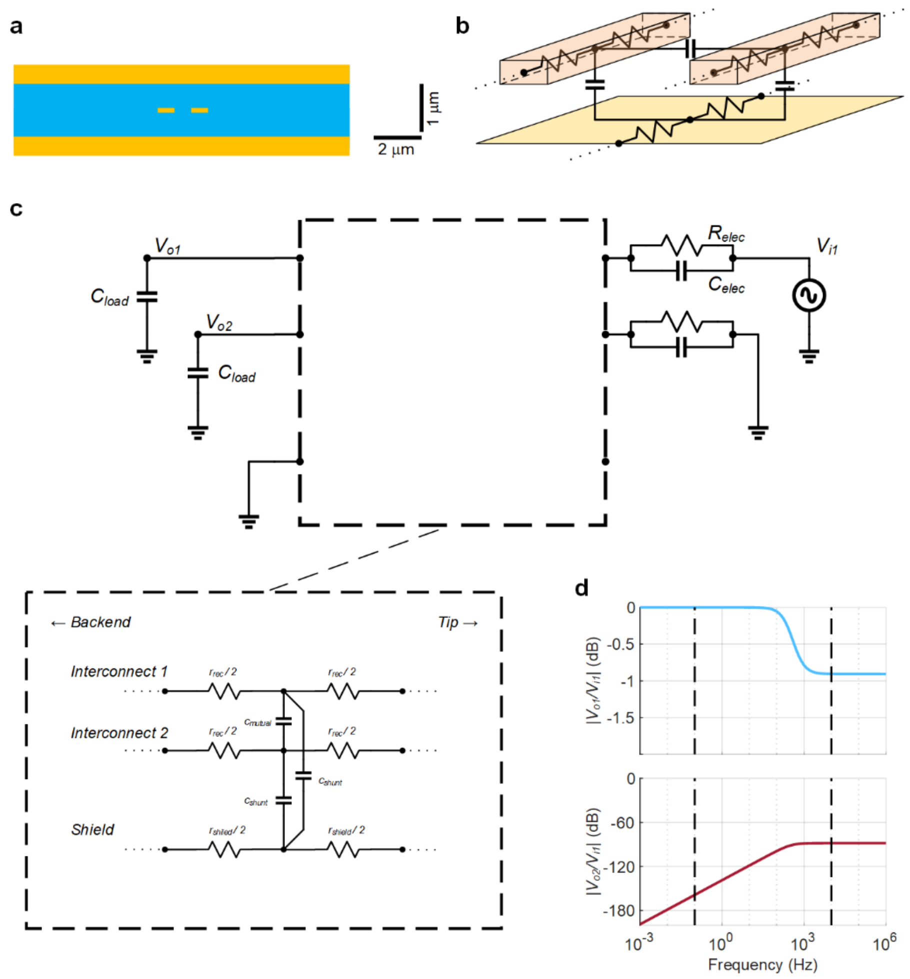

Figure S3. Models used in electrostatic and circuit simulations of the hectoSTAR µLED optoelectrode signal recording circuit. a) Schematic diagram of the cross-sections of a µLED optoelectrode shank, from which the capacitance values were calculated. The width, gap, and the thickness of the interconnects are 700 nm, 700 nm, and 100 nm, respectively. The thickness of the top and the bottom silicon dioxide passivation layers are both 500 nm. b) Schematic diagram of the unit block of the T-network, which represents a 100-µm long shank segment, which was fed into the circuit netlist. Two cuboids indicate the interconnects and the plane at the bottom the (top and bottom) shield layer(s). c) Circuit diagram of a model optoelectrode. The box with dashed lines represents the shank modeled as the T-network. The unit cell of the T-network is shown in the inset. The voltage source representing the neuron, the parallel RC component representing the iridium electrode impedance, and the shunt capacitance representing the amplifier gate capacitance are shown. d) Bode plots of the calculated attenuation and crosstalk of the signal provided to an electrode. Both attenuation (<

1 dB) and crosstalk (< -90 dB) are expected to be minimal throughout the physiologically relevant frequency band (0.1 Hz $\leq$ f $\leq$ 10 kHz).

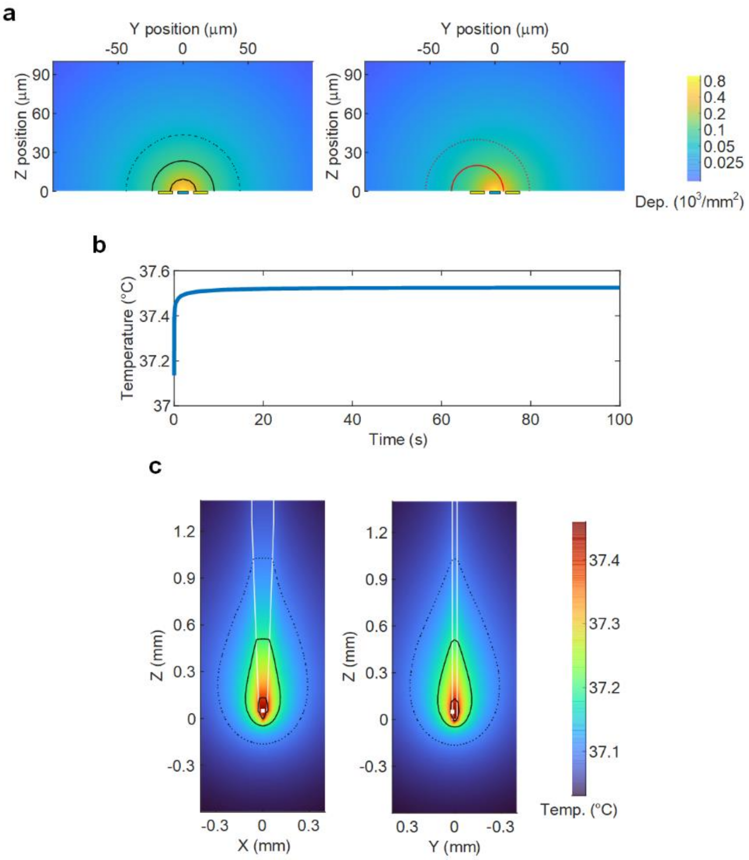

Figure S4. μLEDs on hectoSTAR optoelectrode enable effective and safe optical stimulation of neurons inside a small volume of the brain. a) Simulated profile of the optical flux generated from a μLED on a hectoSTAR optoelectrode. Light blue rectangles indicate the location of the LED, and the yellow rectangles the recordings sites. Black lines are contour lines drawn along the isosurfaces with 0.25 (solid line), 0.1 (dashed line), and 0.05 $mW/mm^2/\mu W$ (dotted line) optical flux. Red lines indicate the isosurfaces of concentric spheres whose centers are located at the center of the recording site and radii equal 20 (solid line) and 40 (dotted line) μm. Note the great overlap between the volume illuminated by the LED light and the volume from which electrical signals are recorded. b) Simulated evolution of maximum temperature on the surface of a hectoSTAR optoelectrode during an optical stimulation with the highest safe power (W = 4 V × 75 μA = 300 μW). The temperature saturates at 37.52 °C. c) Simulated temperature profile of the optoelectrode and the surrounding tissue upon temperature saturation. Locations of the LED utilized are indicated with a white square. Note effective heat dissipation along the optoelectrode shank.

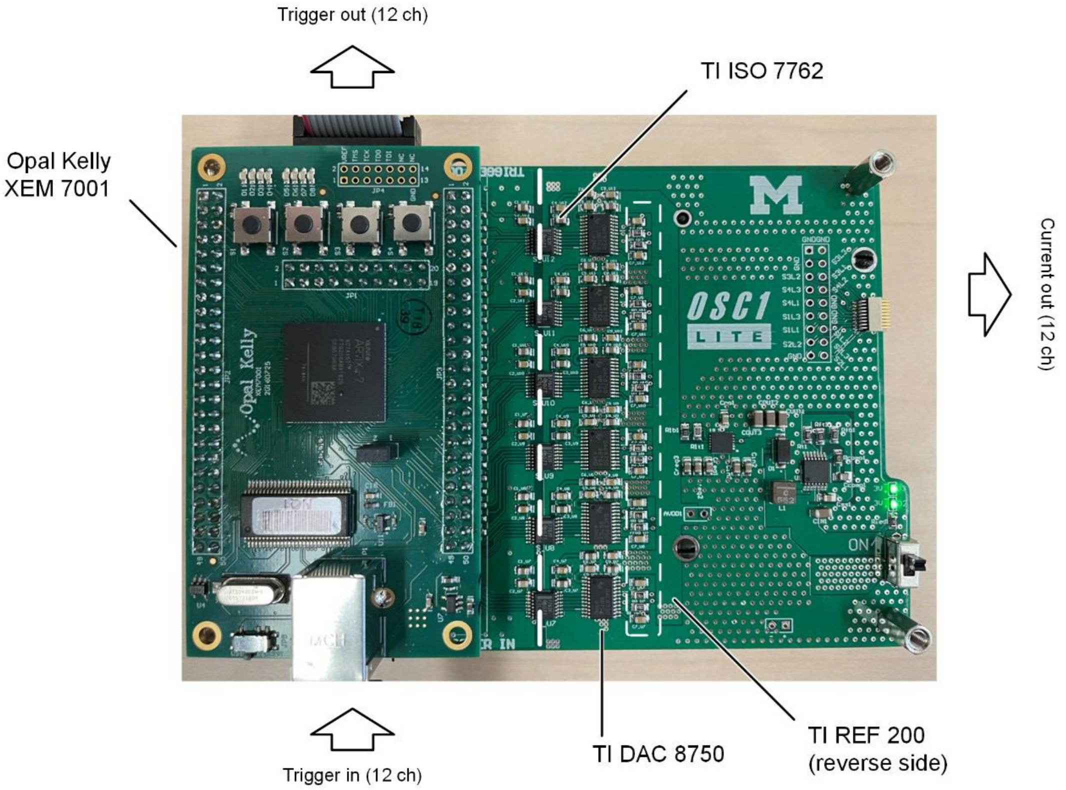

Figure S5. Hardware implementation of OSC1Lite. Photograph of a completely assembled OSC1Lite board. The FPGA-based controller board (Opal Kelly XEM 7001) is shown on the left, and the current digital-to-analog converters (TI DAC 8750, only 6 of which are shown) are located on the right. Along the center of the PCB, TI ISO 7762 digital isolators provide an isolated ground for the current driver domain, in which two 18650 batteries provide 7.4 V DC voltage. Six TI REF 200 current references (12 channels total) are located on the bottom side of the printed circuit board, next to six TI DAC 8750 chips.

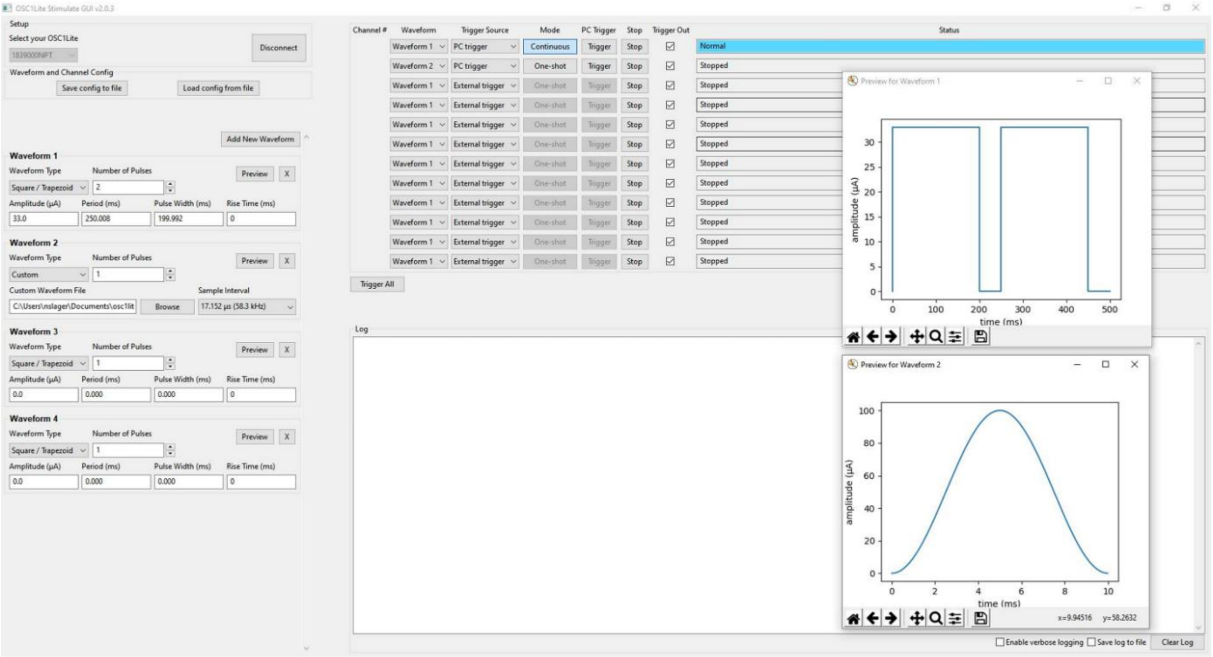

Figure S6. Graphical user interface (GUI) for OSC1Lite. Up to four arbitrary waveforms can be defined and be independently assigned to each current output channel. Users can define waveform type (square, trapezoid, or custom waveform), number of pulses (1-65535), amplitude (peak-to-peak current, 0-100 µA), period (0-17.9 s), pulse width (0-17.9 s) and rise time (0, 0.1, 0.5, 1 and 2 ms). The waveforms can be previewed (top inset show two square pulses, bottom inset show a custom waveform). The GUI can be used to trigger individual or all LEDs with one-shot or in continuous mode. It also provides feedback on the status of an LED (normal, stopped, or open circuit). More details about the GUI can be found on our GitHub (https://raw.githubusercontent.com/YoonGroupUmich/osc1lite/ref200/OSC1Lite_Manual_v2.pdf).

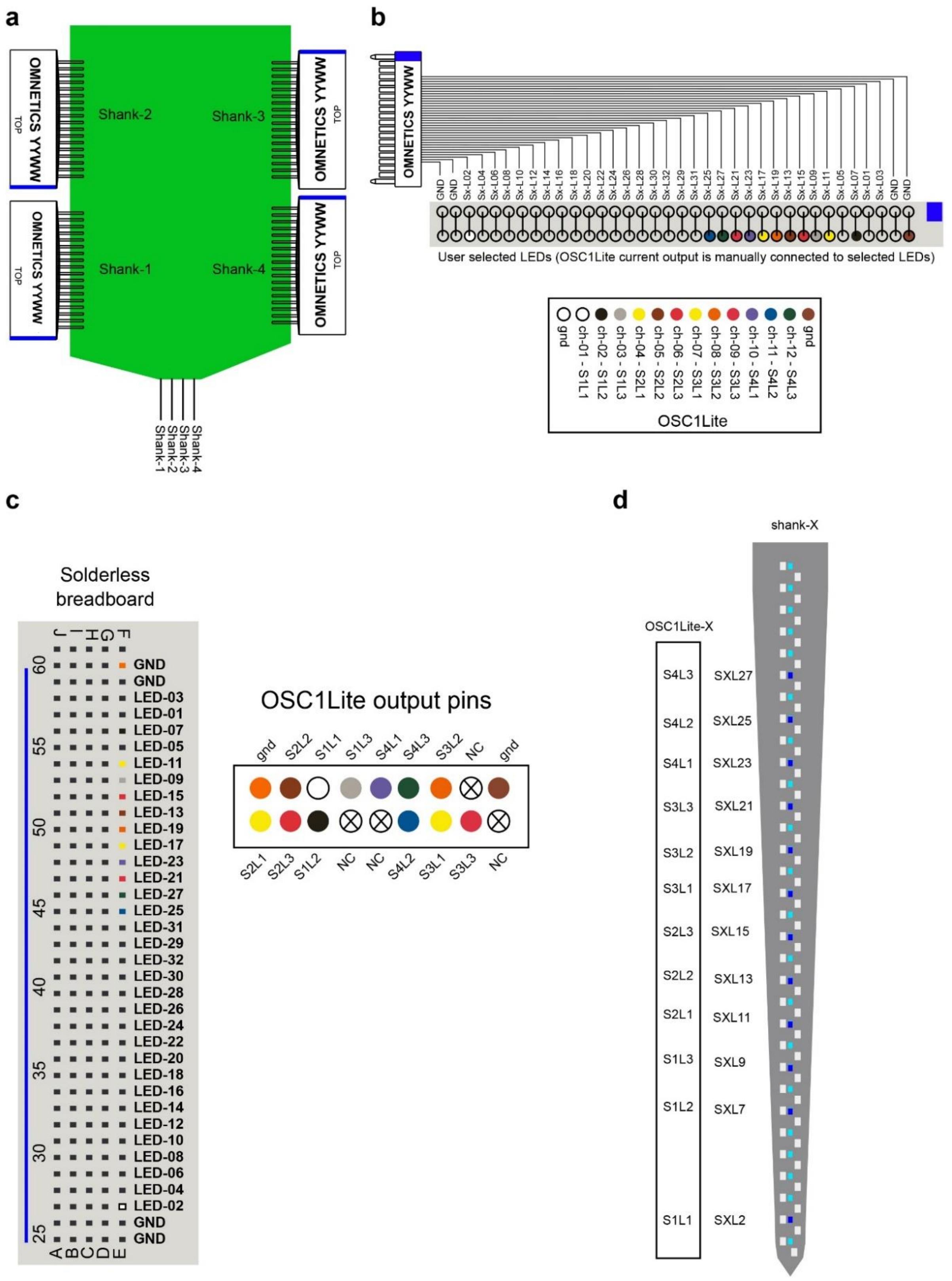

Figure S7. Customized control of µLEDs using OSC1Lite. a) Schematic of probe PCB with four 36-pin Omnetics connector. These connectors are used to control LEDs on each shank. Blue stripes are markers for orientation. b) Male Omnetics connector is attached to the probe PCB and to a solderless breadboard via male header pins. This configuration allows users to

connect the Omnetics connector before starting the experiments and change µLED pins during experiments. Color coded pins represent one of our stimulation configurations. c) Solderless breadboard is shown with 12 µLEDs in use (color coded rectangles, same as in b). Each LED is controlled by a current output of OSC1Lite (F column, same color code scheme). The male header pins of the male Omnetics connector are attached to column G. d) Shank schematic with selected µLEDs is shown from one of our experiments. 12 µLEDs were controlled using OSC1Lite.

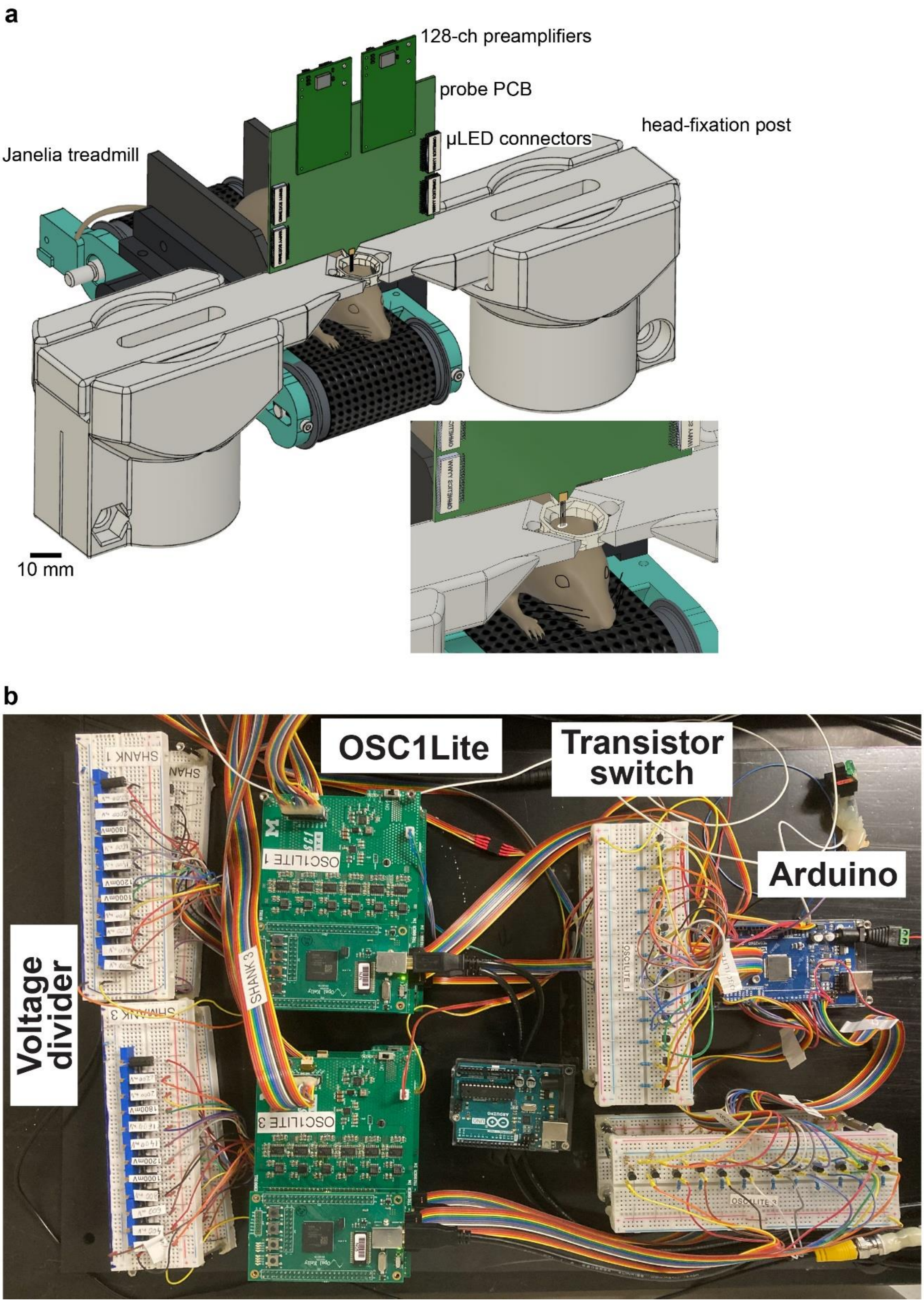

Figure S8. Head-fixed setup for hectoSTAR µLED optoelectrode. a) CAD design of the assembled treadmill system. Mice were head-fixed and were allowed to freely move on the treadmill during recording sessions. A heating-element provided enough comfort for the animals during recording sessions. PCB of the hectoSTAR µLED electrode was held by

alligator clips attached to a manual micromanipulator. Two 128-ch preamplifiers were attached to the probe PCB to record electrophysiology signal. Four 36-pin Omnetics connector were used to deliver current to each μLED. Magnified schematic shows the location of the craniotomy (white circle). b) Photograph of our custom-built circuits used to control hectoSTAR optoelectrode.

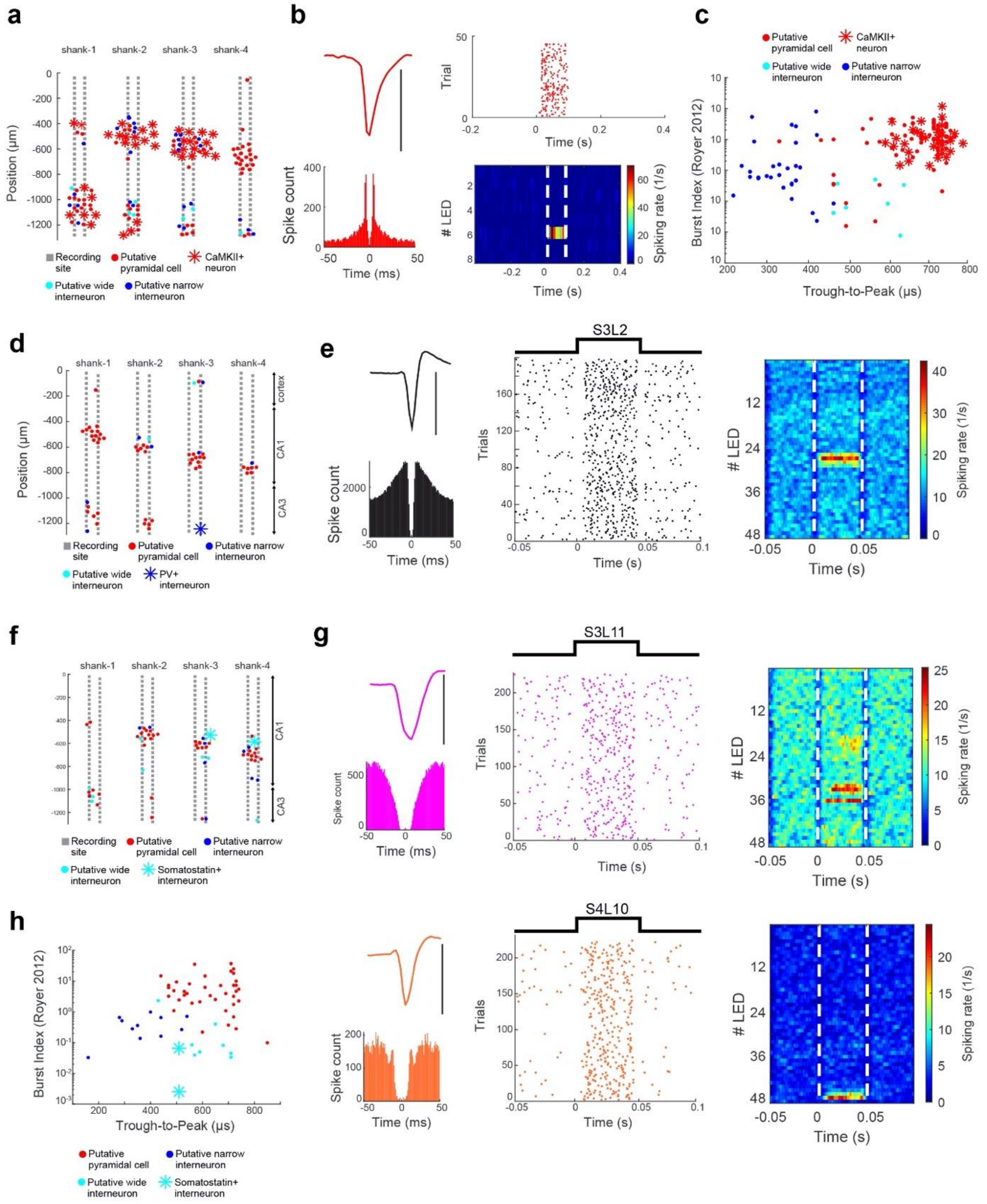

Figure S9. Multi-regional optotagging in head-fixed mice using hectoSTAR μLED optoelectrode. a) Hippocampal (CA1-CA3) recording from a head-fixed mouse (CaMKII-ChR2). Probe layout with the shanks is shown with the putative location of recorded neuron somatas (n = 104 putative pyramidal cells, 29 narrow interneurons and 7 wide interneurons). 45 optotagged cells were recorded in this session (red stars show the putative location of the somatas of these cells). b) Mean waveform and autocorrelation histogram indicate well

isolated single units (top, black scale bar represents 100 µV). Raster plot of the same single unit shows the modulation of spiking activity in response to light stimulation on S3L17 (LED6, n = 45 trials). Right: LED triggered mean spiking rate indicates that the cell was significantly modulated by S3L17 (shank-3 LED17, spiking rate: 28.64 Hz, CI = 0.01-1.64). c) Clustering of neurons by through-to-peak time of their waveform and burst index. d) Hippocampal (CA1-CA3) recording from a head-fixed mouse (PV-ChR2). Optotagging experiments were performed using 48 LEDs (50 ms pulses at 10 Hz, at 40 µA current intensity). Probe layout with the shanks is shown with the putative location of recorded neuron somatas (n = 48 putative pyramidal cells, 8 narrow interneurons and 2 wide interneurons). One optotagged cell was recorded in this session (blue star shows the putative location of the somata of this cell). e) Mean waveform and autocorrelation histogram indicate a well isolated single unit (black scale bar represent 100 µV). Raster plot of the same single unit shows the modulation of spiking activity in response to light stimulation on S3L2 (shank-3 LED-2, n = 198 trials). LED triggered mean spiking rate indicates that the cell was significantly modulated by 4 LEDs (LED25-28, spiking rate: 23.5 Hz, 33.9 Hz, 22 Hz and 17.8 Hz, CI = 7.58-16.16). f) Hippocampal (CA1-CA3) recording from a somatostatin-ChR2 mouse (stimulation parameters were the same as in d). Probe layout with the shanks is shown with the putative location of recorded neuron somatas (n = 45 putative pyramidal cells, 7 narrow interneurons and 9 wide interneurons). Two optotagged cells were recorded in this session (cyan stars show the putative location of the somata of these cells). g) (Left) Mean waveforms and autocorrelation histograms indicate well isolated single units (black scale bars represent 100 µV). (Middle) Raster plots of the same single units show the modulation of spiking activity in response to light stimulation (shank-3 LED-11 for the neuron in magenta and shank-4 LED-10 for the neuron in orange, n = 225 trials). (Right) LED triggered mean spiking rate indicates that both cells were significantly modulated by multiple local LEDs (magenta cell: LED44-46, spiking rate: 5.3 Hz, 10.3 Hz and 16.23 Hz. CI = 1.57-5.2; orange cell: LED31-33 and LED35, spiking rate: 14.6 Hz, 17.9 Hz, 13.2 Hz and 17.7Hz, CI = 7.25-12.74). h) Clustering of neurons by through-to-peak time of their waveform and burst index.

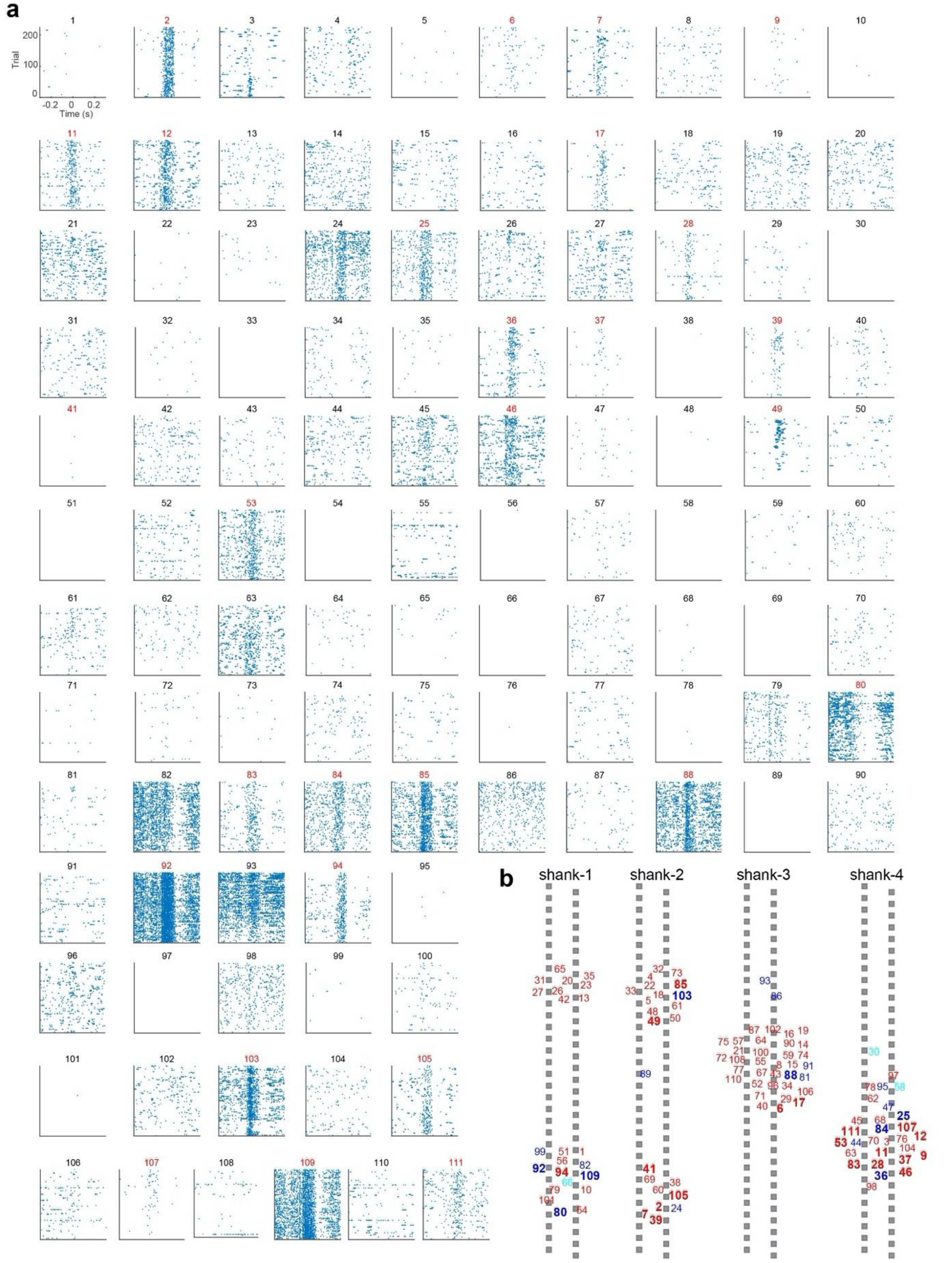

Figure S10. Multi-regional circuit interrogation in a head-fixed mouse using hectoSTAR μLED optoelectrode. a) Well-isolated single units (n=111 putative single units) recorded from the hippocampus (CA1-CA3) of a head-fixed mouse (CAMKII-ChR2). LEDs were activated

in a sequence in CA3 (S1L7, S2L7, S3L2 and S4L1; 120 ms pulses, 15 ms overlap between shanks, 80 µA current intensity). Response of all recorded neurons are shown. Cells in both CA3 and CA1 were affected by the light stimulation, as shown by their raster plots (red numbers indicate significant modulation by light stimulation, bootstrapping parameters: 500 repetitions, CI: 0.001 – 0.999). b) Spatial distribution and putative cell types from the same session as in a. Numbers are closer to the recording site (grey rectangle) at which the largest amplitude waveform was detected. Bold numbers represent the significantly modulated cells (red – putative pyramidal cell, blue – putative narrow interneuron and cyan – putative wide interneuron).

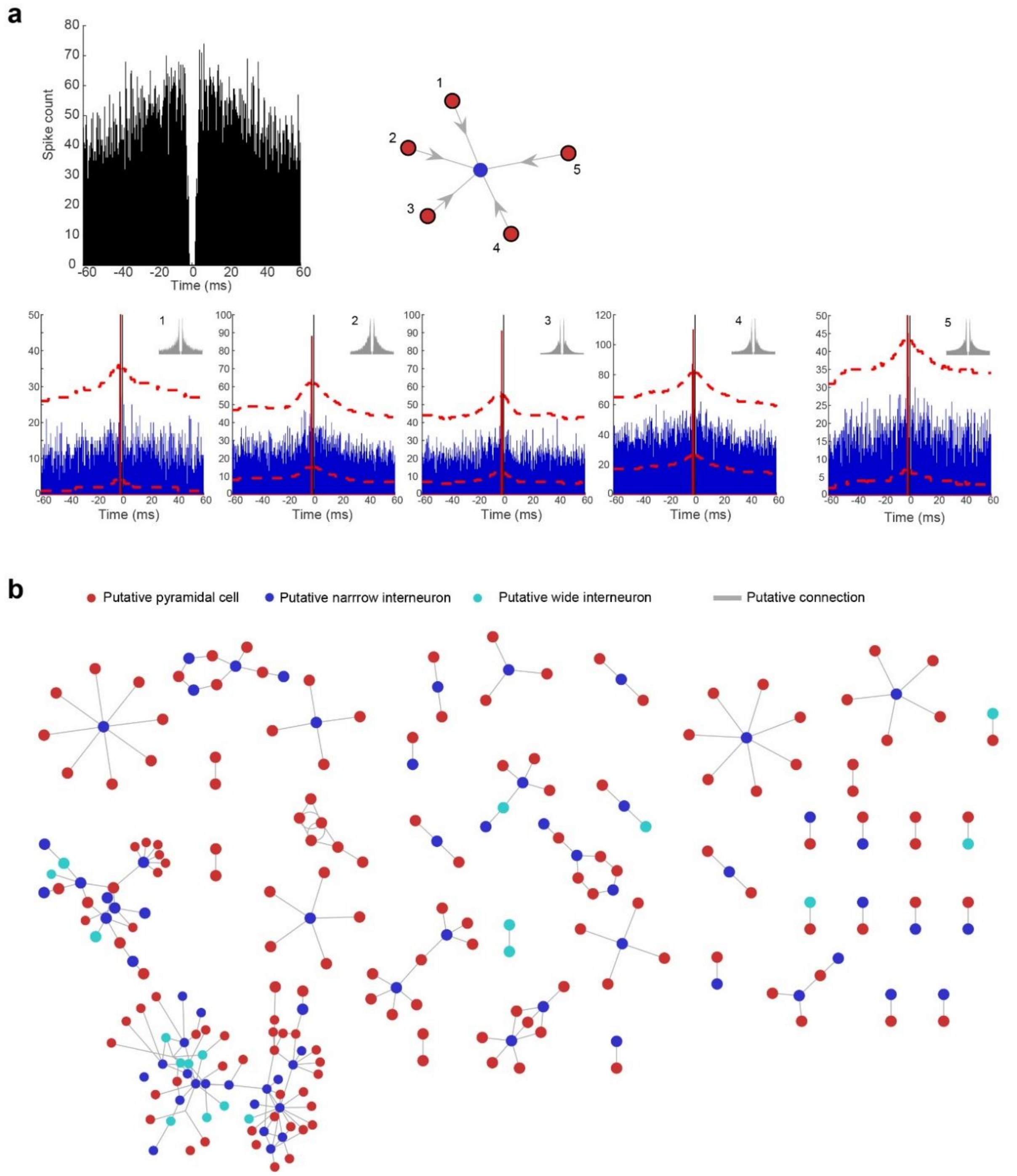

Figure S11. Putative monosynaptic connections between neurons. a) Autocorrelation histogram of a putative narrow interneuron. Cross-correlation histograms (CCG) in blue for five single units (middle black line shows 0 ms lag from the reference spike, dashed red lines represent the confidence interval). CCG binned at 1 ms. Note that all pyramidal neurons have negative (~1 ms) latency peaks (in red) in their pairwise CCGs with the interneuron. b) All detected putative monosynaptic connections in the hippocampus and cortex (n = 231 connections, n = 7 mice).

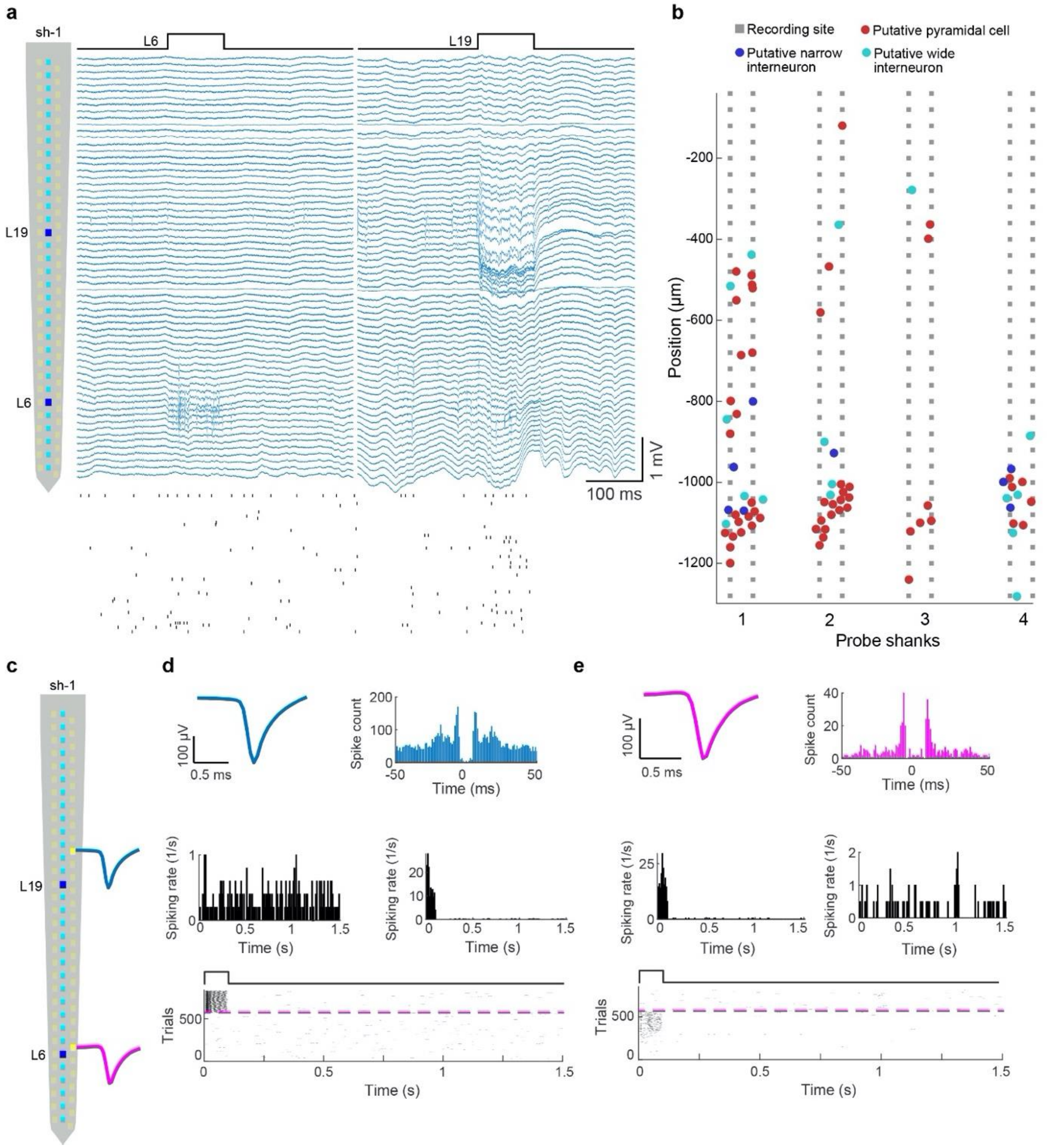

Figure S12. Cortico-hippocampal recordings with hectoSTAR μLED optoelectrode. a) Wide-band signal recorded on 2 shanks (64 channels/shank) during optogenetic stimulation using LED-6 in hippocampus and LED-19 in cortex (L6 and L19, respectively; 100 ms light pulses were delivered at 1 Hz, n = 507 trials in hippocampus and n = 260 trials in cortex) under isoflurane anesthesia in a CaMKII-ChR2 mouse. The location of the active LEDs is shown on the left. Raster plot shows the activity of all active putative single units during μLED stimulation on shank 1. b) Probe layout with the shanks is shown with the putative location of recorded neurons (n = 78 putative single units; n = 54 putative pyramidal cells, 8 narrow interneurons and 16 wide interneurons). Single units are clustered in the cellular layers of

hippocampus and cortex. c) Probe schematic with two example single units (magenta neuron is located in hippocampus, blue neuron is located in cortex). d) Putative pyramidal cell in cortex responds to cortical μLED stimulation only (LED19, spiking rate: 2.85 Hz, CI = 0.23-0.84). Mean waveform, autocorrelation histogram is shown on the top. Peristimulus time histogram of hippocampal and cortical stimulation is shown in the middle (left PSTH is hippocampal stimulation, right PSTH is cortical stimulation). Raster plot shows the trial-by-trial response of the cell. Magenta line represents the change between hippocampal and cortical stimulation. e) Same metrics are shown as in d. The neuron that is located in the hippocampus only responds to hippocampal optogenetic stimulation (LED6, spiking rate: 0.68 Hz, CI = 0.07-0.3).

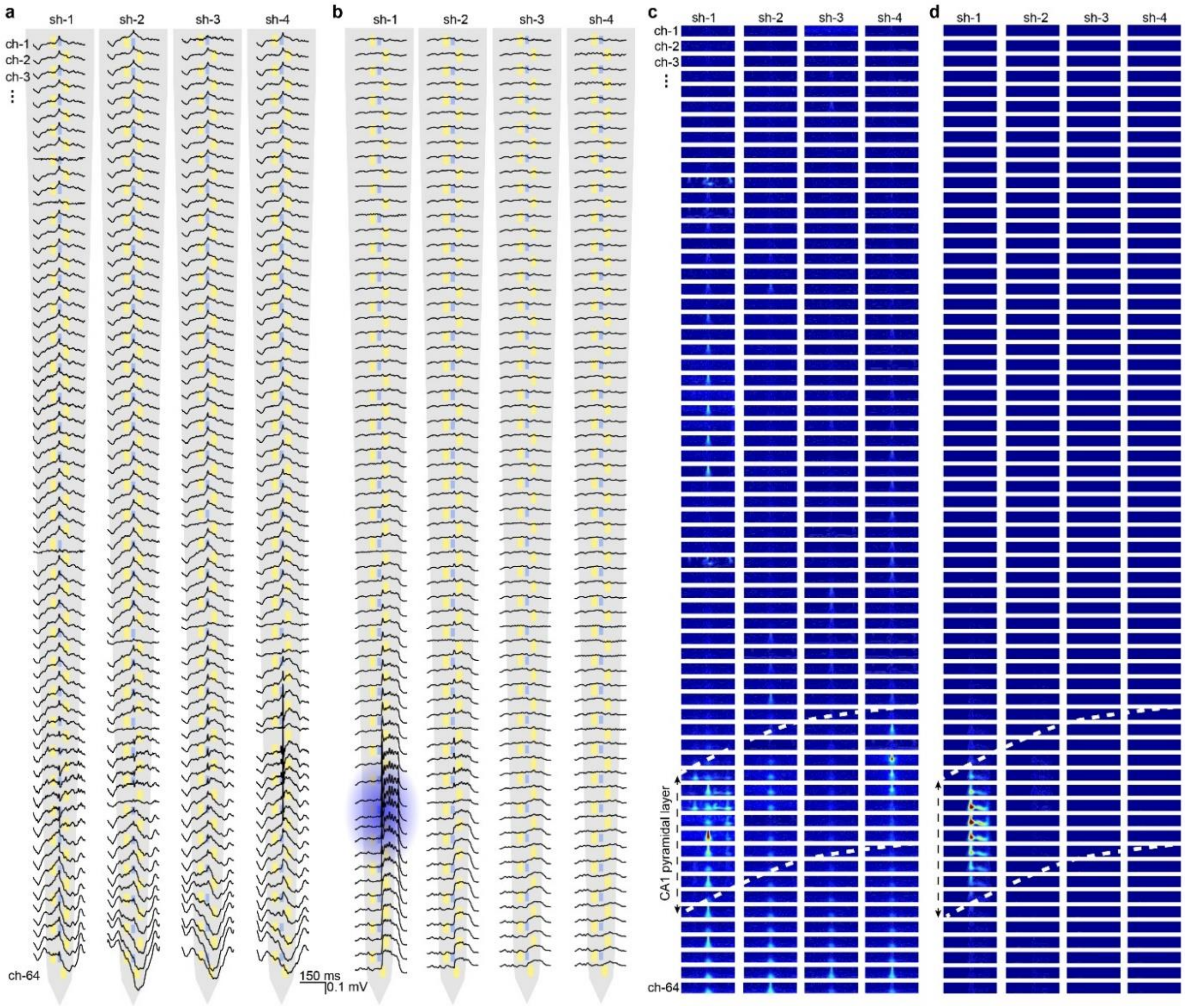

Figure S13. Spatial distribution of spontaneous and induced high-frequency oscillations. a) SPW-R triggered, band-pass filtered average waveforms across all the channels (80-250 Hz band-pass, n = 190 SPW-Rs). b) μLED stimulation triggered, band-pass filtered average waveforms across all the channels. Note, the stimulation-evoked local, high-frequency oscillations in the CA1 region of the hippocampus (100 ms square pulses were delivered on the 6th LED from the tip of shank-1, highlighted in blue; n = 260 pulses at 3.5 V intensity). c) Wavelet spectrograms for spontaneous hippocampal sharp-wave ripples (SPW-Rs). Each panel corresponds to one recording site, arranged from most superficial to deepest, with each column representing one each four shanks of the probe. White dashed lines delineate the hippocampal CA1 pyramidal layer. Above it is the neocortex and below CA1 dendritic layers. Each spectrogram shows the power in the 50-150 Hz frequency band in a window of 300 ms around the SPW-Rs detected in the middle of the pyramidal layer in the leftmost shank (n = 190 SPW-Rs). Note that the characteristic high-frequency power of SPW-Rs is present also in the rightmost shank, 900um away from where they were detected, indicating that SPW-Rs are

synchronous across the whole CA1 covered by the probe. Power in all panels was normalized to the maximum value across all 256 recording sites to facilitate comparison. d) The same spectrograms were calculated around the onset of 100ms square pulse stimulations delivered through a μLED located on shank-1 (6th LED from the tip of the optoelectrode; red asterisk) (n = 260 pulses, 3.5V). Note that the stimulation induced a high-frequency oscillation that resemble the spontaneous SPW-Rs, but in this case, it was restricted to ~ 8 recording sites (160 μm) around the stimulation μLED.

Table S1. Capacitance and resistance values utilized in the electrical circuit simulation of the hectoSTAR μLED. The values for the variables with lower-case names (e.g., r_{rec}) are per-1-mm. Note that the shield layer was effectively treated as a ground, as its sheet resistivity was about three orders of magnitude lower than that of the electrode interconnects.

r_{rec} [Ω/mm]	r_{shield}[a)] [Ω/mm]	c_{mutual} [fF/mm]	c_{shunt} [fF/mm]	R_{elec} [MΩ]	C_{elec} [pF]	C_{load} [pF]
57.1	0	9.44	189	3.01	127	12

[a)] The shield layer was effectively treated as a ground, as its sheet resistivity was about three orders of magnitude lower than that of the electrode interconnects.

Table S2. Values of the parameters utilized in the tissue heating simulation.

Initial and boundary conditions

T_0 [K]	$T_{isotherm}$ [K]
310.15	310.15

Bioheat equation

T_b [K]	$C_{p,b}$ [J/(kg·K)]	ω_b [1/s]	ρ_b [kg/m^3]	Q_{met} [W/m^3]
309.85	3639	0.0085	1060	9700

Material properties

k_{Si} [W/(m·K)]	ρ_{Si} [J/(kg·K)]	$C_{p,Si}$ [kg/m^3]	k_{SiO2} [W/(m·K)]	ρ_{SiO2} [J/(kg·K)]	$C_{p,SiO2}$ [kg/m^3]	k_{brain} [W/(m·K)]	ρ_{brain} [J/(kg·K)]	$C_{p,brain}$ [kg/m^3]
151	2330	724	1.4	2200	730	0.5	1050	3700

Table S3. Stimulation parameters used during experiments of the hectoSTAR μLED.

**Animal ID**	**Active LEDs**	**Active shanks**	**Current [μA]**	**Pulse duration [ms]**	**Pulse period [ms]**	**# trials**	**Brain regions**	**Genotype**
uLED_M01	1,2,3,9,11, 13,15,17,19, 21,23,25	1 to 4	15/20/30/50	100	1000	258	Parietal cortex, CA1, CA3, DG	CamKII-ChR2
uLED_M02	1,2,3,9,11, 13,15,17,19, 21,23,25	1 to 4	15	20	100	414	Parietal cortex, CA1, CA3, DG	PV-ChR2
uLED_M04	1,2,3,5,7,11, 13,15,17,19, 21,23	1 to 4	40	50	100	396	Parietal cortex, CA1	PV-ChR2
uLED_M04	1,2,3,5,7,11, 13,15,17,19, 21,23	1 to 4	40	50	100	198	Parietal cortex, CA1, CA3, DG	PV-ChR2
uLED_M05	7/6/2/2	1/2/3/4	60	120	5000	105	Parietal cortex, CA1, CA3, DG	CamKII-ChR2
uLED_M05	7/7/2/1	1/2/3/4	60	120	5000	224	Parietal cortex, CA1, CA3, DG	CamKII-ChR2
uLED_M06	1,2,3,5,7,11, 13,15,17,19, 21,23	1 to 4	40	50	100	225	Parietal cortex, CA1, CA3, DG	Somatostatin-ChR2
uLED_M07	1/5/2/2 (CA3) 21/21/17/17 (CA1)	1/2/3/4 1/2/3/4	60/80	120	5000	50	Parietal cortex, CA1, CA3, DG	CamKII-ChR2
uLED_M08	3/3/2/2	1/2/3/4	60	100	2000	134	Parietal cortex, CA1, CA3, DG	CamKII-ChR2
Acute_M01	6,19	1	30	200	5000	260	Parietal cortex, CA1	CamKII-ChR2

Table S4. Comparison of some of hectoSTAR μLED optoelectrodes' key feature to those of other multichannel optoelectrodes.

**Publication**	**This work**	**Wu et al. Neuron 2015**	**Kampasi et al. Microsyst. Nanoeng. 2018**	**Libbrecht et al., J. Neurophysiol. 2018**	**Lanzio et al., Microsyst. Nanoeng. 2021**
**No. total recording electrodes (NTRE)**	256	32	32	24	64
**NTRE / shank**	64	8	8	24	64
**Max. inter-electrode distance [μm]**	33.6	53	55	62	40
**No. total stimulation sites (NTSS)**	128	12	4	12	5
**NTSS / shank**	32	3	1	12	5
**Max. no. stim. patterns / shank**	4294967296 (= 2^{32})	8 (= 2^3)	4 (= 2^2) [a]	13 (= ${}_{12}C_1$ + 1) [b]	32 (= 2^5)
**Max. inter-site distance [μm]**	40	60	-	62	150
**No. shanks**	4	4	4	1	1
**Shank cross-sectional area [μm^2]**	144 × 30	70 × 30	70 × 33	100 × 30	45 × 20
**Lithography Technology**	i-line (365 nm)	i-line (365 nm)	Broadband contact	ArF (193 nm)	e-beam
**Light delivery method**	Monolithically integrated LEDs	Monolithically integrated LEDs	On-board diode → monolithically integrated waveguide	On-board diode → monolithically integrated waveguide	External laser sources → optical fiber → monolithically integrated waveguide [c]

[a] Capable of dual-color stimulation from single site; [b] No on-line stimulation multiplexing option available; [c] Multi-wavelength laser sources and a single-mode fiber required.
